# An All‐Soft Wearable Electrochemiluminescence Chip for Sweat Metabolite Detection

**DOI:** 10.1002/advs.202519435

**Published:** 2026-01-04

**Authors:** Wei Nie, Jie Jiang, Xi Wei, Linfeng Zhuo, Danyang Li, Mingyu Jia, Tianhua Zheng, Hua Cui

**Affiliations:** ^1^ Key Laboratory of Precision and Intelligent Chemistry Department of Chemistry University of Science and Technology of China Hefei Anhui P. R. China; ^2^ Department of Biomedical Engineering School of Instrument Science and Opto‐electronics Engineering Hefei University of Technology Hefei Anhui P. R. China

**Keywords:** ECL conductive ionogel, laser‐induced graphene, microfluidics, sweat glucose, wearable sensor

## Abstract

Wearable sensors are transforming real‐time, non‐invasive health monitoring. Despite considerable advances in electrochemical and optical sensing modalities, challenges remain in achieving reliable, sensitive, and cost‐effective detection of sweat metabolites due to the variable chemical composition of sweat and difficulties in device integration. Electrochemiluminescence (ECL) offers an attractive alternative for wearable sensing due to its high sensitivity and ease of integration without complex circuitry or external light sources. However, ECL is rarely used in wearable formats due to challenges in flexible electrode and stable luminophore development. Here, we present a fully soft, skin‐conformable ECL chip for sweat metabolite monitoring, integrating a laser‐patterned closed bipolar electrode array, a microfluidic sweat collector, and deep eutectic solvent‐based ionogels as robust ECL emitters. This architecture separates sensing and reporting zones, effectively reducing signal interference from complex sweat matrices, while the ionogels enhance signal stability and device flexibility, enabling prolonged on‐skin operation. Taking glucose as a model analyte, we demonstrate specific and direct ECL readout by integrating a specific enzymatic reaction with a linear range from µm to mm. The device accurately captures postprandial glucose changes, with a per‐chip cost of $0.106, demonstrating its great potential for real‐time metabolic tracking in personalized health monitoring or clinical diagnosis.

## Graphical abstract

A wearable electrochemiluminescence chip for sweat metabolite monitoring is pioneered, integrating “all‐soft” components: a flexible closed bipolar electrode (c‐BPE) array based on laser‐induced graphene (LIG), a laser‐cut microfluidic layer for sweat collection, and deep eutectic solvents (DES)‐based conductive ionogels for ECL emission.

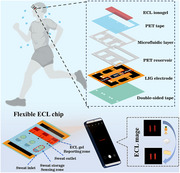

## 1 Introduction

Wearable sensors are rapidly transforming the landscape of non‐invasive health monitoring by enabling real‐time tracking of physiological and biochemical signals directly from the skin [1, 2, 3]. Among various biofluids, sweat is particularly attractive due to its accessibility, continuous secretion, and metabolite‐rich composition [4, 5, 6]. Recently, electrochemical and optical transduction mechanisms have driven much of the progress in wearable sweat sensing [7, 8, 9]. However, stable and reliable monitoring of sweat metabolites remains challenging. Electrochemical sensors are highly susceptible to fluctuations in sweat conductivity, ion composition, and spontaneously active redox species, compromising signal stability and quantification accuracy [1, 10]. In addition, the inherent complexity and high cost of electrochemical acquisition circuits significantly hinder device scalability and widespread clinical adoption [7]. Optical platforms, based on fluorescence (FL) or surface‐enhanced Raman spectroscopy (SERS), typically require external light sources and sophisticated optical acquisition modules, and often suffer from low sensitivity, limited stability, and poor integrability under background interference and mechanical deformation [11]. Therefore, reliable and stable sensing platforms with low cost are highly desired on the skin to avoid the inherent limitations of conventional electrochemical and optical systems, and to fully realize the potential of sweat as a diagnostic and health monitoring medium.

Electrochemiluminescence (ECL) offers a highly promising alternative by directly converting electrical signals into optical readings. Compared to electrochemical sensing, ECL could quantify non‐electroactive targets through catalytic pathways, while avoiding interference from endogenous electroactive species [12]. Furthermore, ECL requires only simple power control to trigger signals, greatly reducing circuit complexity and minimizing potential crosstalk in multi‐point arrays [7]. Importantly, as ECL produces light only during specific redox reactions, it eliminates the need for external illumination and complex optical assemblies, yielding intrinsically low‐background, high‐stability readout, and facile integration compared with FL‐ or SERS‐based optical methods [12, 13, 14]. Despite its widespread success in laboratory diagnostics, ECL remains largely unexplored in wearable platforms due to multiple challenges: (1) Conventional ECL setups depend on bulky equipment such as potentiostats and photomultiplier tubes [15, 16]. (2) The reported ECL sensors lack integrated fluidics for autonomous sweat sampling and instantaneous detection. (3) Conventional ECL luminophores need to react in a liquid conductive medium, which is unstable on flexible, curved surfaces. (4) Moreover, the processing capacity of traditional ECL sensors is one sample per test, involving electrode connections and repeated reagent replacements. Although ECL array electrodes have been reported to improve detection throughput, their fabrication mainly relies on rigid electrode photolithography or printing, limiting miniaturization, cost‐effectiveness, and flexibility [17, 18, 19].

**FIGURE 1:**
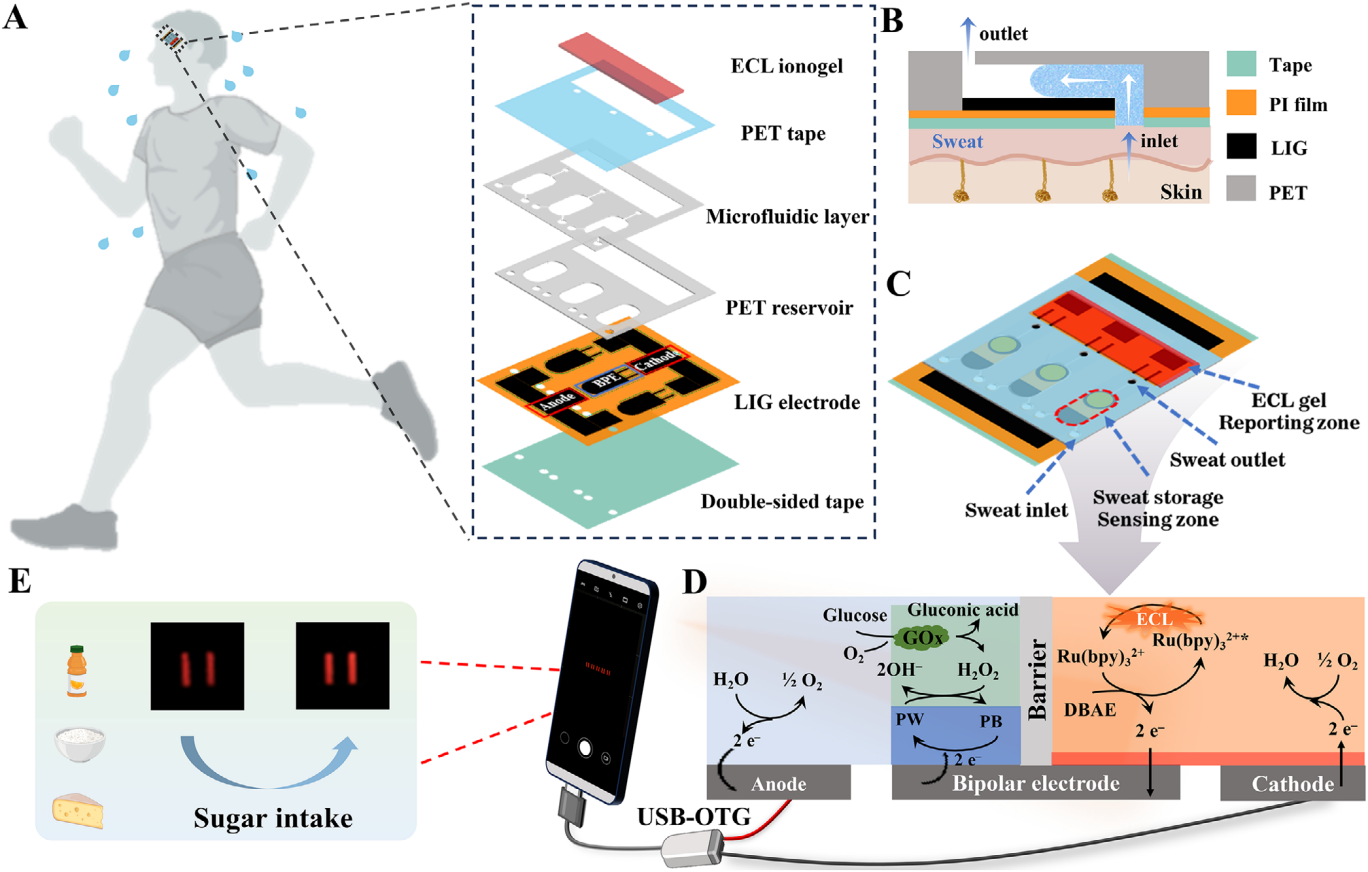
Schematic diagram of the microfluidic flexible ECL array chip for sweat glucose detection. (A) Layer assembly of the flexible wearable ECL chip: The bottom layer consists of a double‐sided adhesive tape for epidermal attachment. The second layer incorporates the LIG flexible electrode. The third layer features a PET reservoir housing the functional materials (PEDOT‐PB, GOx‐CS, and ECL ionogel). The fourth layer is a microfluidic chip designed to collect sweat and deliver it to the sensing zone via capillary action. (B) Schematic cross‐section of the microfluidic chip for sampling sweat secreted by human sweat glands. (C) Schematic of the complete flexible ECL sensing chip. (D) Principle of the ECL sensing chip for glucose detection. (E) Dual function of smartphone: for ECL chip powering and capturing ECL images.

To address these, we pioneered a wearable ECL chip for sweat metabolite monitoring, integrating “all‐soft” components: a flexible closed bipolar electrode (c‐BPE) array based on laser‐induced graphene (LIG), a laser‐cut microfluidic layer for sweat collection, and deep eutectic solvents (DES, a mixture of choline chloride (ChCl), ethylene glycol (EG), and urea in a 1:2:1 molar ratio)‐based conductive ionogels for ECL emission (Figure 1A–C). The spatial separation between the sensing and signal‐reporting zones, enabled by the c‐BPE architecture, minimizes matrix interference from complex sweat constituents, ensuring accurate metabolite detection under on‐skin operation. Simultaneously, DES gel was innovatively used as an ECL luminescent matrix to develop a stable, skin‐compatible, flexible solid‐state ECL ionogel, enabling the chip to detect analytes using only collected sweat samples without external liquid reagents. This simplifies system design and eliminates the risk of reagent leakage, further promoting its suitability for wearable applications where skin conformity and reliability are crucial. As a proof‐of‐concept, we further designed and integrated Poly(3,4‐ethylenedioxythiophene)‐Prussian blue (PEDOT‐PB) and glucose oxidase‐chitosan gel (GOx‐CS)‐modified cathodic BPE arrays for sweat glucose monitoring (Figure 1D). This configuration enabled specific and direct ECL transduction of sweat glucose levels, with signal visualization and analysis performed via a smartphone, which also served as the portable power source (Figure 1E). Notably, postprandial increase in sweat glucose was directly detected, demonstrating the reliability and flexibility of the constructed wearable ECL chip. The proposed ECL flexible sensor chip holds significant potential for real‐time monitoring of multiple sweat metabolites, advancing applications in health monitoring, medical diagnostics, and clinical research.

## 2 Results and Discussion

### 2.1 Designing LIG‐Assisted c‐BPE Array Chip for Accurate ECL Signal Readout

**FIGURE 2:**
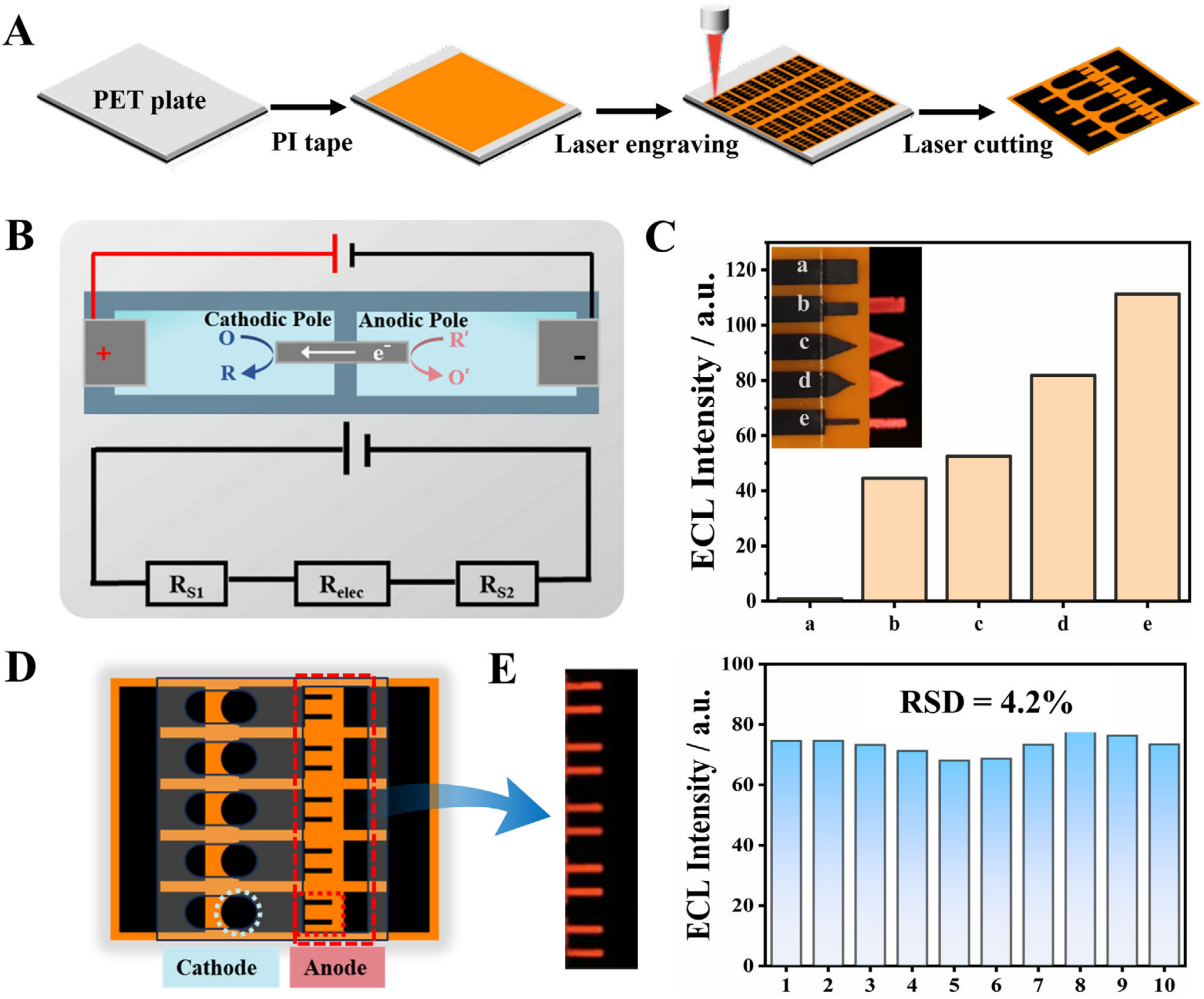
Construction and shape optimization of LIG‐BPE array electrodes. (A) Schematic diagram of the fabrication process of LIG array electrodes. (B) Schematic principle and equivalent circuit diagram of c‐BPE. (C) Comparison of corresponding ECL intensities for BPE anodes with different shapes. Inset: Photographs of BPE anodes with different shapes and corresponding ECL images. (D) Schematic diagram of the designed LIG‐BPE array chip. (E) ECL image and intensity statistics correspond to the designed LIG‐BPE array chip.

Due to the complex composition of sweat and the possible presence of environmental contaminants, employing a conventional three‐ or two‐electrode system, where sensing and signal reading occur in a single working area, may compromise the accuracy and stability of the ECL signal readout. To reduce such interference, a closed bipolar electrode (c‐BPE) system that physically separates the cathode and anode chambers was employed, enabling independent reduction and oxidation reactions upon electrical activation (Figure 2B). In the c‐BPE‐ECL system, molecular recognition occurs at the cathode, while ECL emission is localized at the anode. According to the principle of bipolar electrochemistry, changes in charge transfer at the sensing interface—induced by target binding—are mirrored by corresponding changes in the ECL signal at the reporting site [20, 21].

To construct a flexible, high‐performance c‐BPE array, we utilized laser‐induced graphene (LIG), which offers excellent electrical conductivity, high surface area, and rapid, mask‐free patterning capabilities [22, 23]. As shown in Figure 2A, by using infrared CO_2_ laser irradiation, polyimide (PI) tape adhered to a polyethylene terephthalate (PET) substrate was selectively carbonized via a photothermal process to form LIG embedded within the PI matrix [24]. The output power of the laser engraving machine was optimized at 5.4 W (Figure S1). This approach enabled the fabrication of a precise and flexible electrode array directly onto soft substrates (detailed characterization provided in the Section S2.1 and Figure S2).

To further enhance ECL intensity and improve sensing accuracy to facilitate straightforward visual readout, we systematically optimized the geometry of the BPE anode using the classical Ru(bpy)_3_^2+^/TPA ECL system. Initial experiments optimized the resistance of the cathode‐anode connection section of the BPE. The results demonstrated a linear decrease in ECL intensity with increasing resistance (R = 0.983, Figure S3), which was attributed to the large resistance reducing electron transfer efficiency at the BPE electrodes, thereby limiting the Faraday current. A 2 mm connection width was selected to balance high signal strength with compact chip dimensions. Next, shape optimization was performed by varying the end angle (Figure S4) and area (Figures S4 and S5) of the BPE anode. The experimental results showed that the anode with the sharpest top angle and the smallest area obtained the highest ECL signal. This phenomenon was attributed to an increase in current density (J) with decreasing anode area. The ECL intensity (p) is described by the relationship p~(1/A)⋅(dn_p_/dt), where the photon count (n_p_) is proportional to the number of electrons transferred at the electrode [25, 26]. Therefore, as the current density J (J = I/A) increased, the ECL intensity was enhanced. The reduction in anode area achieved by narrower rectangular or higher skeletonization led to higher current densities and stronger ECL intensity. Finally, to comprehensively evaluate the influence of shape and area on the ECL intensity of BPE anodes, the different anode designs, as shown in Figure S4, were integrated into a comparative study to identify the optimal shape (inset of Figure 2C). The results demonstrated that the elongated rectangle, which had the smallest area dimension with a width of 0.4 mm, produced the strongest ECL signal. Interestingly, even among anodes with identical areas—such as the 1/2 rectangular (b), 40°‐pointed (c), and writing‐brush‐shaped (d) designs—ECL intensity progressively increased with decreasing top angle. This outcome can be attributed to the localized increase in current density at sharper top angles, which enhances ECL intensity.

Thus, a comparative study of various anode geometries (Figure S4, inset Figure 2C) identified the elongated rectangular shape (2 mm length × 0.4 mm width) as the optimal design, delivering the strongest ECL signal. To implement this at the array level, we fabricated a five‐unit LIG‐BPE chip (Figure 2D). Each BPE anode consisted of two elongated rectangles to provide mutual reference points for signal calibration, while the cathode was designed as a semicircle to facilitate integration with a reagent reservoir for functional modification. The resulting LIG‐BPE array exhibited excellent signal uniformity, with consistent ECL emission across the ten active sites and a relative standard deviation (RSD) of just 4.2% (Figure 2E), confirming high fabrication reproducibility and signal reliability.

### 2.2 Developing Flexible ECL Conductive Ionogel for Wearable Sensors

Conductive ionogels with good mechanical properties, reliable electrical conductivity, and stability have been widely used in flexible devices [27, 28]. DES is a low‐melting‐point eutectic mixture of hydrogen bond donors and acceptors, which has the advantages of environmental friendliness, low cost, low volatility, and good ionic conductivity, and has now become an ideal alternative to ionic liquids in traditional ionogels [29]. However, DES‐based ECL gels have rarely been reported. Here, we report a flexible, ECL‐active ionogel composed of a ternary DES‐based electrolyte, gelatin as the polymer matrix, Ru(bpy)_3_^2+^ as the luminophore, and DBAE as the co‐reactant. Ternary DES consists of ChCl, EG, and urea in a molar ratio of 1:2:1, whose composition and characterization are discussed in detail in Section S2.3 and Figure S6. Gelatin, a biocompatible polymer, promotes strong hydrogen bonding, hydrophobic, and electrostatic interactions with DES components, ensuring a stable and homogenous gel network [30]. Furthermore, DBAE, an environmentally friendly co‐reactant, demonstrates superior performance in the Ru(bpy)_3_^2+^/DBAE system compared to the Ru(bpy)_3_^2+^/TPA system [31].

**FIGURE 3:**
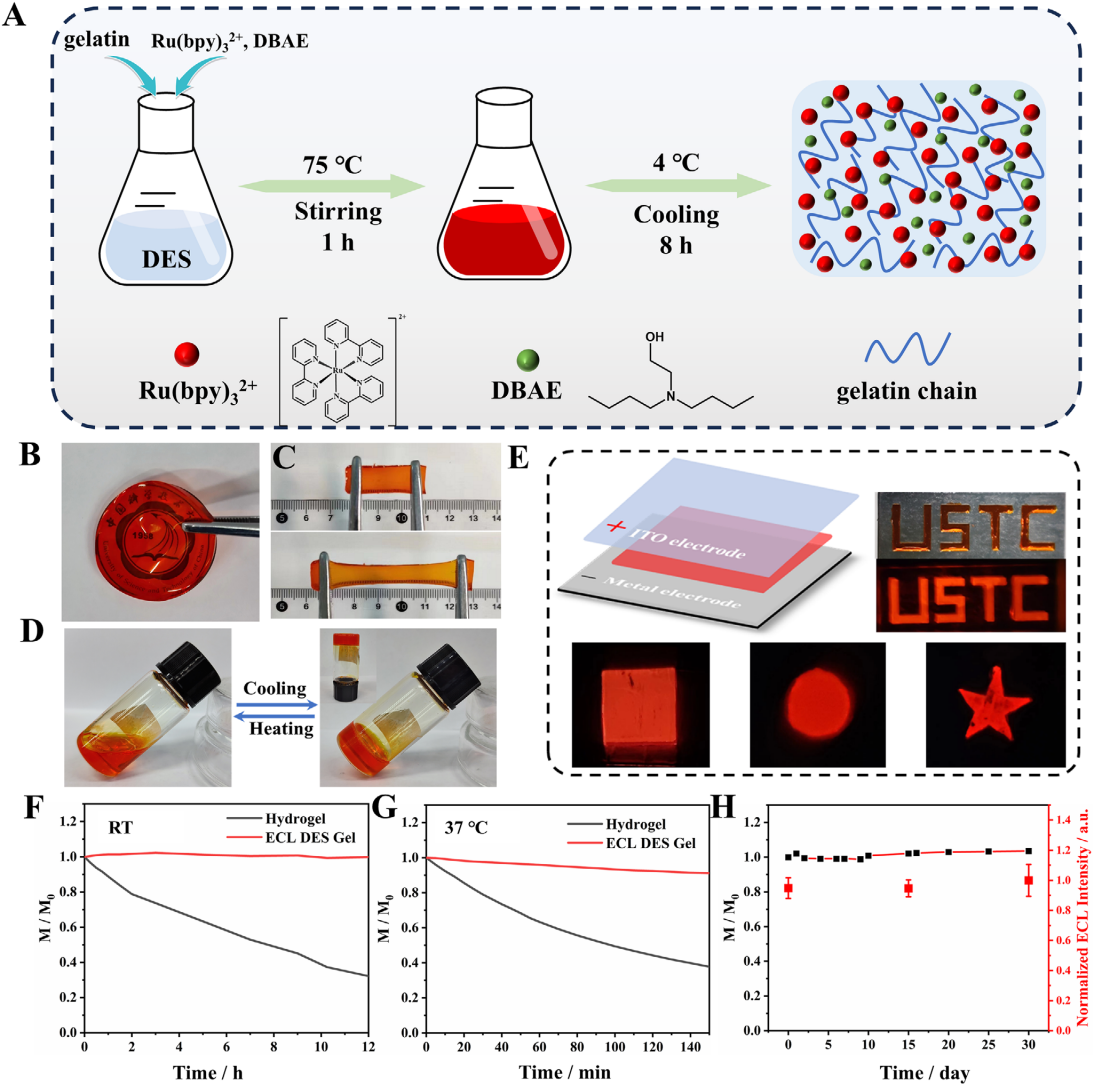
Synthesis method of ECL conductive ionogel and its properties. (A) Schematic of the synthesis of the ECL conductive ionogel. Light permeability (B), stretchability (C), and its state after heating and cooling (D) of the synthesized ECL conductive ionogel. (E) Pictures of ECL emission when the ECL conductive ionogel was sandwiched between two electrodes and energized with DC power. DC voltage optimized to 2.8 V (Figure S10). Mass change of gelatin‐based hydrogel (black curve) and ECL DES gel (red curve) with time at room temperature (F) and 37°C (G). (H) Changes in the mass and ECL intensity of the prepared ECL gel within one month.

The synthesis process of the ECL conductive ionogel is shown in Figure 3A. When the mixture of DES, gelatin, Ru(bpy)_3_^2+^, and DBAE was heated, it transformed into a clarified solution, which solidified into the desired ECL gel upon cooling. The resulting ECL gel exhibited essential properties for flexible device construction, including good light permeability (Figure 3B), stretchability (Figure 3C), elasticity (Figure S7B), adhesion (Figure S8), and conductivity (4.07 mS·cm^−1^, Figure S9). Additionally, the gel demonstrated reprocessing capability, allowing conversion between liquid precursor and solid gel states through repeated heating and cooling cycles (Figure 3D; Figure S7A). Moreover, the efficient ECL‐emitting molecules and co‐reactants endowed the conductive ionogel with good ECL performance, which could produce bright ECL emission when sandwiched with two electrodes and applied with a suitable DC voltage (Figure 3E). DES‐based ionogels exhibited excellent thermal stability and ultra‐low volatility, enabling prolonged device usage under ambient conditions without additional sealing or encapsulation. This property was a key advantage over hydrogels. As shown in Figure 3F, a comparison of weight loss between the ECL DES gel and hydrogel under room temperature storage conditions revealed that the hydrogel lost approximately 68% of its water content within 12 h, while the ECL DES gel showed negligible weight loss. At 37°C, the hydrogel retained only 37.8% of its original weight after 2.5 h, compared to the ECL DES gel, which maintained 91.2% of its weight (Figure 3G). Furthermore, the weight and ECL intensity of the ECL DES gel remained virtually unchanged after one month of storage at room temperature (Figure 3H).

These findings highlighted the superior non‐volatility of DES and its excellent compatibility with gelatin scaffolds, both of which were crucial for the long‐term performance of ECL DES gel‐based devices under ambient conditions. Finally, the ECL mechanism of the synthesized ECL conductive ionogels was investigated and explored in detail in the Section S2.5, Scheme S1, and Figure S11.

### 2.3 Construction of Flexible ECL Chip with High Stability

**FIGURE 4:**
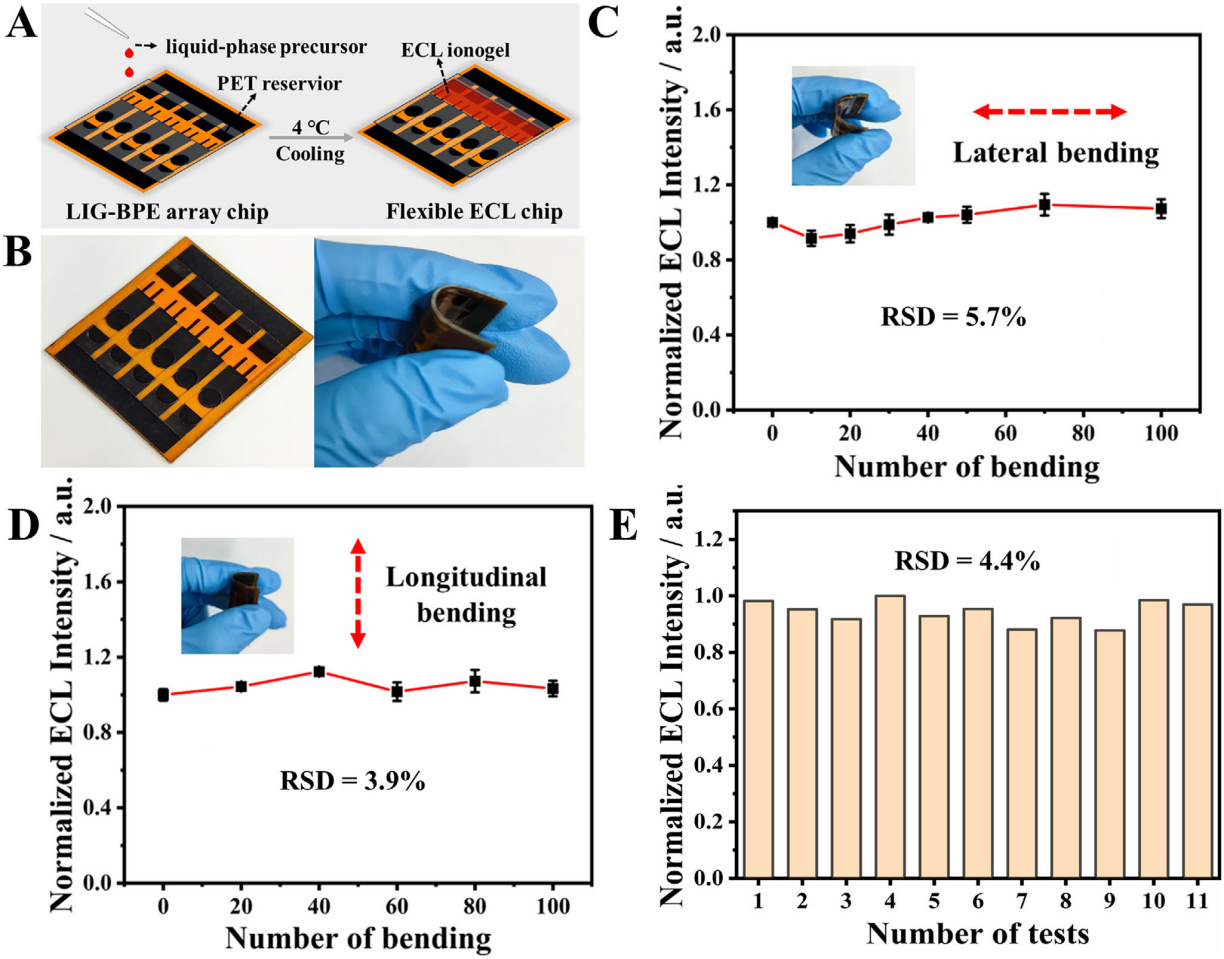
Construction of the flexible array chip and its stability investigation. (A) Schematic diagram of ECL gel‐modified LIG‐BPE array microarray construction. (B) Physical image of ECL gel‐modified LIG‐BPE array chip. Changes in ECL intensity of ECL gel‐modified LIG‐BPE array chip with 100 times of lateral (C) and longitudinal (D) bending. (E) ECL intensity variation of the constructed array chip was tested 11 times in a row. The error bars represented the standard deviation of three independent replicates.

By integrating the LIG electrodes and ECL conductive ionogels, we first developed a flexible chip that can be adapted to a curved interface. The prepared ECL gel liquid‐phase precursor was introduced into the reporting zone of a flexible LIG‐BPE array chip. After cooling and gel molding, the ECL ionogel‐modified LIG‐BPE array chip was successfully fabricated (Figure 4A). The gel adhered firmly to the LIG‐BPE array chip, remaining intact even when the chip was subjected to bending deformation (Figure 4B). This behavior mirrored the way wearable devices adapt to epidermal deformation, ensuring consistent functionality during mechanical strain.

To assess the stability of the flexible device during deformation, the ECL gel‐modified LIG‐BPE array chip underwent 100 cycles of both lateral and longitudinal bending, with a bending angle exceeding 60° in each instance. The ECL intensity was recorded throughout the bending process. As shown in Figure 4C,D, the ECL intensities remained essentially unchanged after repeated lateral and longitudinal bending, with relative standard deviations (RSD) of 5.7% and 3.9%, respectively. These results confirmed the strong and stable modification of the ECL gel on the LIG‐BPE array chip. Furthermore, the chip's stability was validated through 11 consecutive voltage applications. The ECL intensity remained consistent, with an RSD of 4.4% (Figure 4E). This further demonstrated the reliability and robustness of the developed array chip for potential applications in flexible and wearable sweat metabolite sensing devices.

### 2.4 Glucose Sensing Using PEDOT‐PB/GOx Modified LIG‐BPE Array

Given the clinical significance of glucose and its strong correlation between sweat and blood concentrations [32, 33, 34], we selected sweat glucose detection as a model to evaluate the performance of the flexible ECL array chip. In the closed bipolar electrode (c‐BPE) system, the principle of electroneutrality enables electron transfer between spatially separated cathodic (sensing) and anodic (reporting) zones, thereby translating biochemical recognition into amplified ECL signals.

**FIGURE 5:**
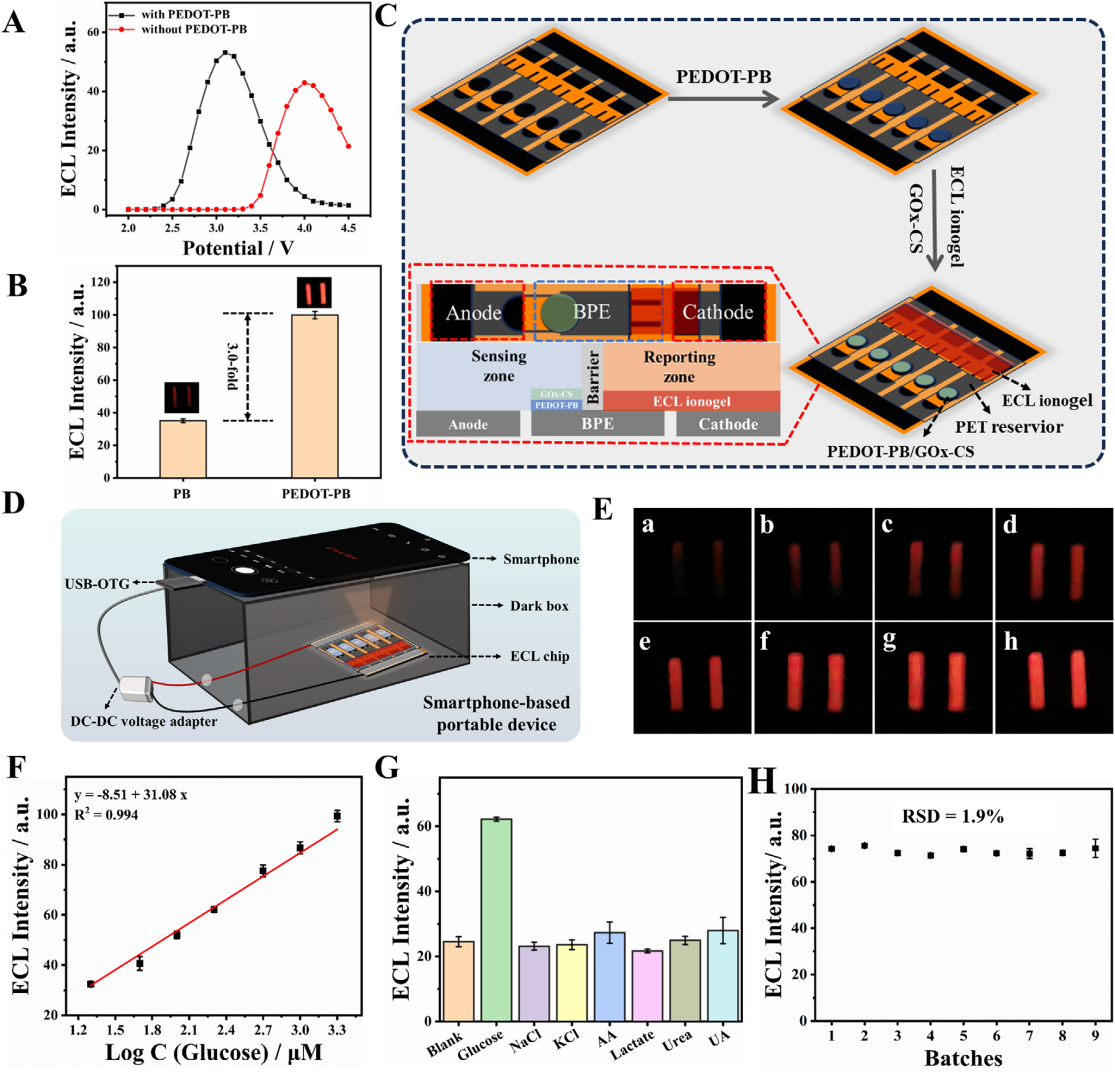
Flexible ECL array chip construction for glucose detection and its analytical performance. (A) ECL‐potential curves of the constructed array chip, modified and unmodified PEDOT‐PB. (B) ECL intensity of the constructed array chip modified with PB or PEDOT‐PB. (C) Schematic diagram of flexible glucose sensing array chip construction. (D) Schematic of smartphone imaging platform for glucose detection. (E) ECL images of glucose with different concentrations (a–h): 0, 20, 50, 100, 200, 500, 1000, and 2000 µm. (F) Relationship curve between the ECL intensity and the logarithm of glucose concentration. (G) Selectivity of the constructed glucose sensing chip: blank, glucose (0.2 mm), NaCl (100 mm), KCl (40 mm), AA (0.2 mm), lactate (40 mm), urea (20 mm), and UA (0.2 mm). (H) ECL intensity of the fabricated glucose sensor at different batches (C_glucose_ = 0.5 mm). The error bars represented the standard deviation of three independent replicates.

To enhance the electrocatalytic response, Prussian Blue (PB) was employed as a redox‐active mediator due to its peroxidase‐like activity toward hydrogen peroxide (H_2_O_2_), a byproduct of glucose oxidation by glucose oxidase (GOx) [35]. However, PB's poor conductivity limits its performance, prompting the integration of the conductive polymer PEDOT to form PEDOT‐PB nanocomposites [36]. Figures S12 and S13 in Section S2.6 provide detailed synthesis and characterization of PEDOT‐PB. These materials offered improved redox kinetics and conductivity, as characterized in Section S2.7 and Figure S14. When a PBS solution containing 1 mm H_2_O_2_ was introduced into the reporting zone of LIG‐BPE, the PEDOT‐PB‐modified chip exhibited lower onset and peak potentials alongside stronger emission (Figure 5A). These enhancements were attributed to the high electrocatalytic activity and low reduction potential of PEDOT‐PB. The reduced onset potential was critical for real sample detection because it minimized the occurrence of side reactions. Furthermore, at the optimized detection voltage of 2.6 V, the anodic ECL intensity of the PEDOT‐PB‐modified chip was three times higher than that of a chip modified with PB alone (Figure 5B), unequivocally demonstrating the superior redox properties and electrocatalytic performance of PEDOT‐PB nanocomposite. In the cathodic sensing zone of LIG‐BPE, PEDOT‐PB was applied as the first layer, serving as an efficient electrocatalyst, while GOx‐CS formed the second layer as a specific enzyme to catalyze glucose oxidation (Figure 5C). According to the principle illustrated in Figure 1D, the addition of glucose to the sensing area triggered rapid oxidation catalyzed by GOx, resulting in the production of H_2_O_2_. PEDOT‐PB, with its high electrocatalytic activity, catalyzed the reduction of H_2_O_2_ upon energization. This reaction enhanced electron transfer, leading to a significant amplification of the ECL signal in the anodic reporting zone of the prepared ECL ionogel.

Under optimal experimental conditions (Figure S15), the analytical performance of the developed ECL conductive ionogel‐based LIG‐BPE array chip for glucose detection was further evaluated. The analytical setup consisted of a minimal configuration, including an array chip, a 3D‐printed dark box, and a smartphone, making it portable, cost‐effective, and user‐friendly (Figure 5D). A smartphone could both capture images and also be used as a power source to output a stable voltage via the USB‐On The Go (USB‐OTG) reverse charging function (Figure S16). Figure 5E illustrates the ECL signal images corresponding to varying glucose concentrations in a 1×PBS solution added to the sensing zone of the array chip. A clear increase in ECL intensity was observed with increasing glucose concentrations. In the range of 20−2000 µm, the ECL intensity exhibited a strong linear relationship with the logarithm of glucose concentration, yielding a correlation coefficient of R^2^ = 0.994 (Figure 5F). The observed linear relationship between ECL intensity and logarithmic glucose concentration arises from multi‐step cascading reactions and synergistic interactions (enzymatic, PEDOT‐PB electrocatalysis, and BPE signal transduction). Multi‐step signal transduction is typically rate‐limited, yielding non‐linear distributions across broad concentration ranges; systems involving enzymes or catalytic saturation naturally produce linear calibration curves on a logarithmic scale [37, 38]. Additionally, the detection range of the chip sensor almost covers that of sweat glucose concentrations (from µm to nearly mm level) from healthy individuals to those with pathological conditions reported in the literature [39, 40, 41, 42, 43, 44]. This also ensures that the sensor remains unsaturated under physiological conditions and can track the full range of glucose dynamics. To verify the activity of GOx modification in the sensing zone, the post‐reaction solution was tested with the Diammonium 2,2’‐azino‐bis(3‐ethylbenzothiazoline‐6‐sulfonate) (ABTS)/horseradish peroxidase (HRP) mixture. As demonstrated in Section S2.11 and Figure S18, only the solution resulting from the glucose reaction in the chip's reporting zone and the H_2_O_2_ solution could cause the ABTS/HRP mixture to turn green, supporting that glucose successfully converted to H_2_O_2_ through enzymatic reaction in the reporting zone. The distinct color change served as direct visual proof of the successful enzymatic reaction and the generation of H_2_O_2_, thereby confirming the bioactivity of the immobilized GOx layer. The sensor demonstrated a limit of detection (LOD) of 14.3 µm (S/N = 3), surpassing the performance of several previously reported wearable glucose sensors [44, 45, 46, 47, 48, 49].

To evaluate the selectivity of the ECL sensor, interference tests were conducted using three key types of interfering substances documented in the literature, including electrolytes (NaCl, KCl), easily oxidizable small molecules (ascorbic acid (AA), uric acid (UA)), and high‐abundance metabolites (lactate, urea) [50, 51, 52, 53]. Their concentrations approached or exceeded the physiological upper limits for interfering substances [54, 55], thereby validating the sensor's specificity under extreme conditions. As shown in Figure 5G, even under such stringent testing conditions, the chip exhibited a significant ECL signal response only to glucose, with minimal interference from other substances, which was attributed to the physical isolation provided by the c‐BPE architecture, the high electrocatalytic activity of PEDOT‐PB toward H_2_O_2_, and the low applied voltage. Reproducibility tests across different batches indicated consistent performance. For a glucose concentration of 0.5 mm, the RSD of ECL intensity across nine parallel experiments was 1.9% (Figure 5H), confirming the sensor's high reproducibility. Stability tests showed minimal ECL intensity changes over three weeks when sensors were stored at 4°C (RSD = 5.36%). After three weeks, the ECL intensity retained 83% of its initial level, demonstrating good long‐term stability (Figure S17). The practical application of the sensor for detecting glucose in artificial sweat samples was evaluated using standard addition methods. Tests in artificial sweat revealed that ECL intensity increased with glucose concentration, maintaining a strong linear relationship (Figure S19). Notably, ECL intensities and slopes were slightly higher in artificial sweat compared to PBS, likely due to the higher ionic concentration and favorable pH in artificial sweat [56]. In real sweat spiking experiments, glucose concentrations of 61.1 and 109.3 µm were detected in two samples, with spiking recoveries ranging from 94.9% to 109.1% (Table S1). These results indicated that the flexible ECL array chip is suitable for analyzing glucose concentrations in real sweat.

### 2.5 Direct Sweat Glucose Detection by the Wearable ECL Sensor

**FIGURE 6:**
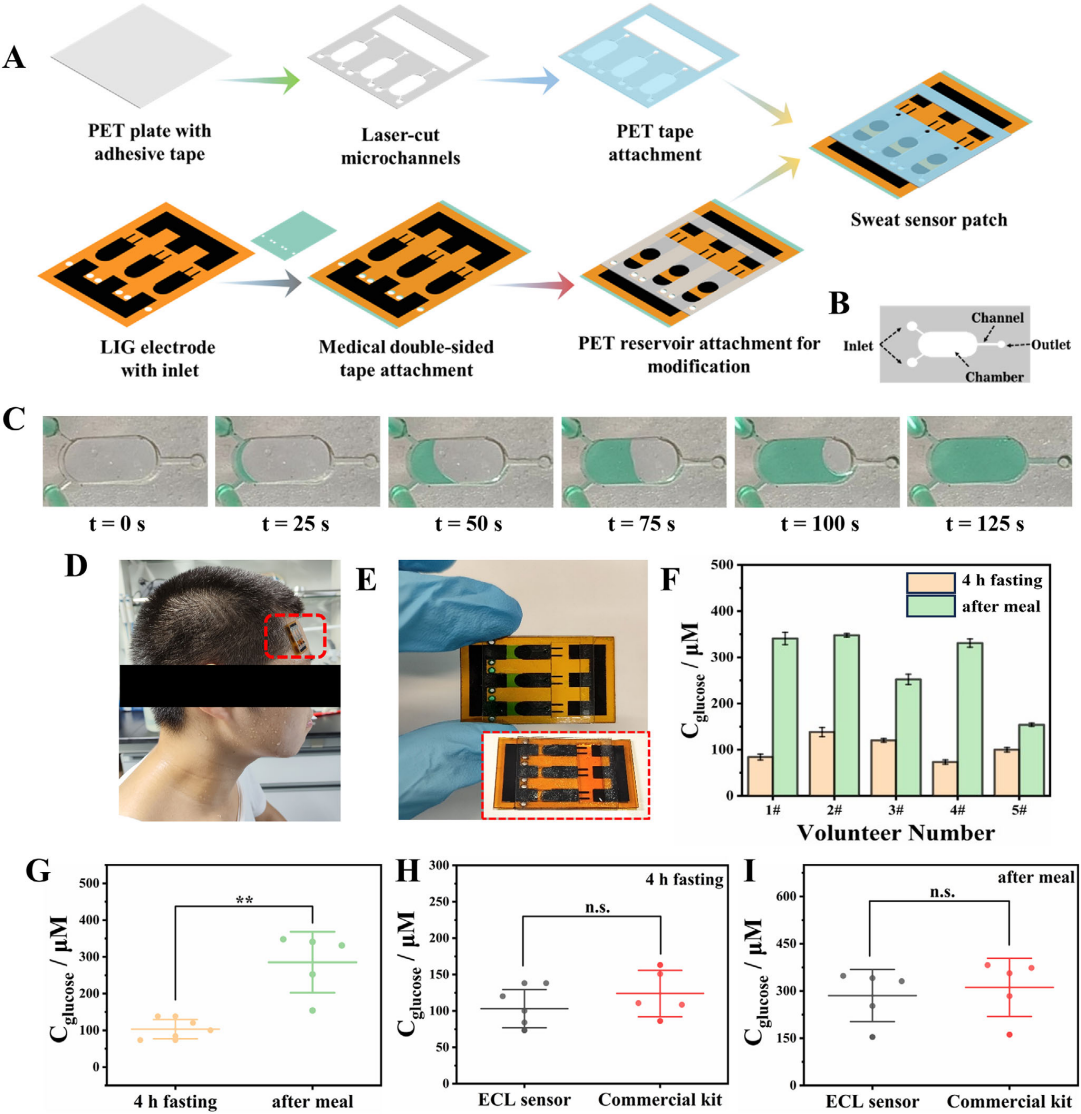
The constructed ECL wearable sensing array chip with a microfluidic patch for in vitro evaluation of sweat glucose. (A) Schematic diagram of the construction steps. (B) Schematic structure of the microfluidic cell. (C) Physical diagrams of the green dye flow through the microfluidic chip chamber at different times (flow rate: 5 µL/min). (D) Photograph of a wearable ECL patch mounted on the forehead of a volunteer after strenuous exercise. (E) Physical images of the flexible ECL chip before and after dye adsorption in the sensing zone. (F) Sweat glucose concentrations after 4 h of fasting and after meals in 5 volunteers were tested by the wearable ECL sensor. The error bars represented the standard deviation of three independent replicates. (G) Scatter plots and significance analysis of sweat glucose concentrations in volunteers after 4 h of fasting and after meals. Scatter plots and significance analyses of sweat glucose concentrations after 4 h of fasting (H) and after a meal (I) in volunteers tested with the developed wearable ECL sensor and commercial kit. Statistical significance was calculated via the T‐test. n.s., not significant; ^*^ *p* < 0.05, ^**^ *p* < 0.01.

To enable automated sweat collection for wearable detection, a microfluidic patch was designed and fabricated through laser cutting, which allowed for low‐cost and rapid batch manufacturing. As shown in Figure 6A, the patch was assembled using multiple pre‐designed patterned PET layers. The microfluidic layer included two sweat inlets, a sweat storage chamber aligned with the sensing zone of the ECL chip, and a sweat outlet (Figure 6B). To reduce the chip size while maintaining sufficient parallel tests, the throughput of each ECL array chip was reduced from five to three units. Detailed dimensions were provided in Figure S20. After plasma treatment of the microfluidic chip, a green dye solution was introduced into the inlet to visualize the liquid flow. The dye rapidly filled the sweat storage chamber, as shown in Video S1. To further observe liquid dynamics within the transparent microfluidic chip, a syringe pump was used to inject green dye at a controlled flow rate of 5 µL/min. The chamber gradually filled with the dye, reaching full capacity at approximately 125 s (Figure 6C; Video S2). The collected sweat volume was calculated to be about 20 µL, consistent with the results shown in Figure S21A. Notably, once the chamber was filled, the liquid remained intact due to the narrow channel design, providing sufficient buffer time for subsequent ECL measurements (Figure S21B). Additionally, green dye droplets were placed in a petri dish to simulate sweat samples. When the sensing zone of the microfluidic patch was placed on the droplets, the dye quickly and autonomously filled the sensing chamber (Video S3), demonstrating the capability of the patch to collect sweat independently and effectively.

A wearable ECL sensor patch for human in vitro sweat glucose detection was developed by integrating a microfluidic chip with a flexible ECL chip. On‐body testing of the wearable patch was conducted by attaching it to the forehead of a volunteer after strenuous exercise, allowing for sweat collection and enzymatic glucose oxidation reactions (Figure 6D). The physical appearance of the wearable patch is shown in Figure 6E, where the retained green dye in the sensing zone highlights its capability for automated dye collection. Sweat glucose tests were performed on five volunteers, both after 4 h of fasting and after meals, using the wearable ECL patch. Quantitative results, presented in Figure 6F, showed that sweat glucose concentrations were significantly lower after fasting compared to postprandial levels. Individual differences in glucose concentration changes were observed; for instance, Volunteers 1# and 4# exhibited substantial differences, while Volunteer 5# showed relatively smaller changes. Statistical analysis revealed significant differences in sweat glucose concentrations between the fasting and postprandial states for all participants (*p* < 0.01, Figure 6G), confirming that glucose levels in human sweat have been altered by meal consumption. To evaluate the accuracy of the sensor, the results were compared with those from a commercial enzyme‐based glucose assay kit, quantified via spectrophotometry (Figure S22). As shown in Figure 6H,I, no significant differences were found between sweat glucose concentrations measured by the wearable ECL sensor and the commercial kit for both fasting and postprandial states, which demonstrated the high accuracy of the developed wearable ECL sensor. These findings highlighted the potential of the novel wearable sensor based on ECL conductive ionogel and LIG flexible array chip for in vitro sweat glucose detection. Moreover, the developed ECL sensing chip employs a disposable design that offers key advantages for wearable sensing applications, including eliminating cross‐contamination risks, ensuring each measurement starts from a pristine and consistent sensing interface, and greatly simplifying the user operation by eliminating cleaning or recalibration requirements. The low production cost of the array chip—only $0.106 per chip (Table S2)—positioned it as a cost‐effective solution for wearable glucose sensing applications.

## 3 Conclusion

In summary, we have developed a flexible, wearable ECL array chip that represents a significant step forward in the application of ECL technology for non‐invasive sweat metabolite detection. The device integrated a laser‐induced graphene‐based closed bipolar electrode (LIG‐BPE) array, a DES‐based ECL conductive ionogel, and a microfluidic sweat collection patch into a compact, flexible platform. This fully integrated system enabled spatially separated sensing and reporting, stable signal output, and mechanical adaptability suitable for on‐skin operation. Taking glucose detection as a model application, the capability of the wearable sensor has been demonstrated, which allowed the selective glucose sensing with high sensitivity (LOD = 14.3 µm), reproducibility, and long‐term storage stability. Real‐sweat tests further validated its ability to track physiologically relevant changes in metabolite concentration, such as postprandial glucose variation. Signal acquisition and power supply via a smartphone further streamlined the platform, supporting portable and user‐friendly operation.

This work establishes a foundation for ECL‐based wearable sensors capable of detecting a broad range of sweat metabolites. Compared with electrochemical wearable sensors, our proposed ECL chip with c‐BPE‐ECL architecture not only converts electrochemical signals into optical readings but also decouples the reaction and reporting zones, effectively mitigating matrix interference without complex circuitry. Relative to FL‐ or SERS‐based optical sensors, the absence of an external light source results in lower background interference and improved integrability. Together with its low cost (∼$0.106 per chip), high sensitivity, and mechanical robustness, this wearable ECL platform provides a promising route toward real‐time, non‐invasive health monitoring and clinic diagnosis.

Future efforts will focus on the development of miniaturized imaging devices compatible and adaptable to the human epidermis for sensitive and multiplexed detection of metabolites coupled with artificial intelligence (AI)‐driven data analysis. The blood‐sweat correlation will also need to be investigated. These developments will accelerate clinical translation of wearable sweat‐sensing technologies and will support data‐driven healthcare and clinical decision‐making, thereby promoting preventive medicine and remote diagnostics.

## 4 Experimental Section

### 4.1 Construction of LIG‐BPE Array Electrodes

The fabrication process of the LIG‐BPE array electrodes is as follows. Initially, Kapton polyimide (PI) tape was applied evenly to a polyethylene terephthalate (PET) substrate. This PI/PET composite structure (total thickness ∼175 µm, with PI ∼75 µm and PET ∼100 µm) was selected over a standalone PI film to ensure a flat, wrinkle‐free surface for uniform laser patterning, to provide robust mechanical support preventing curling or fracture of the flexible electrode during handling, and for its cost‐effectiveness. The PI/PET surface was then cleaned by rinsing with ethanol and deionized water, followed by nitrogen blow‐drying. The prepared PI/PET substrate was placed in a CO_2_ laser cutting and engraving machine. Using a pre‐designed computer program, the patterns for the BPE electrodes were imported, and the corresponding LIG‐BPE array electrodes were produced in engraving mode. The laser engraving machine parameters were set as follows: engraving speed of 150 mm/s, stepwise resolution of 0.05 mm, and optimized output power of 5.4 W (Figure S1). Finally, the monolithic array electrodes were processed in cutting mode to create individual electrodes and sweat feed ports. Additional precision cuts were made to form the sweat inlets on the electrodes and to finalize the electrode pieces.

### 4.2 Preparation of ECL Conductive Ionogel

The ternary deep eutectic solvent (DES) was prepared according to a previously reported method [57]. Choline chloride (ChCl), ethylene glycol (EG), and urea were mixed in a molar ratio of 1:2:1 and heated at 80°C with constant stirring until a clear liquid was formed. The prepared DES was sealed and stored for later use. For the synthesis of the ECL conductive ionogel, gelatin, Ru(bpy)_3_Cl_2_·6H_2_O, DBAE, and DES were added to a reaction flask at a mass ratio of 14:0.2:0.4:86. The mixture was sealed and placed in a water bath at 70°C with continuous stirring for over 1 h until a transparent and homogeneous solution was obtained. After cooling, this solution served as the ECL gel precursor. The final ECL gel was formed by transferring the ECL precursor solution into a mold, sealing it with Parafilm, and storing it in a 4°C refrigerator overnight. Before characterization or further testing, the gel was equilibrated to room temperature after removal from the refrigerator.

### 4.3 Fabrication of LIG‐BPE Glucose Sensing Chip

The LIG‐BPE glucose sensing chip was constructed according to the following steps. First, a laser‐cut PET reservoir was affixed to a pre‐prepared LIG‐BPE array chip, in which the individual sensing regions were isolated and independent of each other. Then, 6 µL of PEDOT‐PB aqueous solution was added dropwise to the BPE cathode in each sensing zone. Next, the ECL gel precursor solution was added to the reporting zone of the LIG‐BPE array in a 45°C incubator and maintained for 20 min to allow the liquid to spread out evenly. Subsequently, 6 µL of a mixture (1:1, v/v) of 10 mg/L GOx (dissolved in 0.01 m PBS, pH = 7.2) and 2.5 wt.% CS solution was added dropwise to the PEDOT‐PB‐modified BPE cathode. Finally, the modified BPE electrode array was dried in a 4°C refrigerator overnight to obtain a LIG‐BPE glucose sensing chip, in which the BPE cathode was modified by PEDOT‐PB/GOx‐CS, and the reporting zone was modified by ECL ionogel.

### 4.4 Glucose Detection with LIG‐BPE Flexible Chip

For the test of standard concentration glucose, 20 µL of different concentrations of glucose solutions dissolved in 0.01 m PBS solution were added to the sensing zone of the constructed LIG‐BPE sensing chip separately and allowed to react for 5 min at room temperature. Then, two pairs of magnets were used to clamp the positive and negative poles of the BPE electrodes and connect them to the power supply. The array chip was placed in a 3D‐printed dark box (17 × 10 × 11 cm) to block any external light source. Finally, the power was turned on, and the ECL images were captured using a smartphone in its professional mode with an exposure time of 2 s. The ECL intensity of the captured images was analyzed using ImageJ software. The collected sweat tests and spiked recovery experiments followed the same procedure.

### 4.5 On‐Body Implementation of the Wearable ECL Chip

Prior to on‐body testing, a microfluidic module was fabricated using a CO_2_ laser cutter to facilitate the flow of sweat from human skin into the sensing chip. The cutting speed was set to 30 mm/s, and the output power was 8.2 W. First, the sweat inlets were cut out of the designed LIG‐BPE chip, ensuring the inlets were free of any engraved LIG through a rational design. Then, medical double‐sided tape with inlets (adhesive layer) was aligned and affixed to the bottom of the LIG‐BPE chip. Next, PET tape with sweat inlets, outlets, and modification zones was laser‐cut and attached as the second layer. The third layer consisted of a microfluidic channel, and the fourth layer was a sealer with a sweat outlet, both prepared from laser‐cut PET sheets with adhesive tape (thickness: 0.1 mm). The microfluidic modules underwent plasma treatment prior to testing to enhance their hydrophilicity. For each sensing unit, there were two sweat inlets, an ellipse‐like reservoir, and an outlet. Detailed dimensions are provided in Figure S20. With ethical approval from the Medical Research Ethics Committee of the First Affiliated Hospital of the University of Science and Technology of China (Number: 2024‐N(H)‐309, 2025KY478), five healthy volunteers (3 males and 2 females, aged 23–28) with no relevant medical history were selected as subjects for on‐body sweat glucose testing. Written informed consent was obtained from all participants prior to the study commencement. They fully consented to demonstrate the wearable ECL sensor and release the results publicly. After 30 min of strenuous exercise, a prepared wearable ECL chip was attached to the forehead of each volunteer, allowing sweat secreted by the skin to fill the sensing area within 2 min through the microfluidic channels. Glucose levels in sweat were determined by ECL intensity captured through smartphone photos. Additionally, the sweat glucose levels of the volunteers were compared after 4 h of fasting and post‐meal consumption. All volunteers consumed a sugary beverage with their meal and underwent a sweat glucose test 20 min afterward. For comparison, a commercial test kit (JL‐T1042, Jianglai Biology, Shanghai) was used to test the collected sweat samples.

### 4.6 Statistical Analysis

All statistical plots in the work were represented by means ± standard error of the mean (s.e.m.), scatter plots, bar charts, or scatter interval, drawn by Origin software (version 9.0). Data normalization was represented by dividing each data point by the mean of the corresponding dataset. The error bars represented the standard deviation of three independent replicates. The t‐test was used for two‐group comparisons. All statistical analysis were carried out with the Prism software package (PRISM 8.0; GraphPad Software). The threshold for statistical significance was defined as *P* < 0.05.

## Conflicts of Interest

The authors declare no conflicts of interest.

## Supporting information




**Supporting File 1**: advs73670‐sup‐0001‐SuppMat.docx.

**Supporting File 2**: advs73670‐sup‐0002‐VideoS1.mp4.

**Supporting File 3**: advs73670‐sup‐0003‐VideoS2.mp4.

**Supporting File 4**: advs73670‐sup‐0004‐VideoS3.mp4.

## Data Availability

The data that support the findings of this study are available from the corresponding author upon reasonable request.
