# Supplementary material for: An All‐Soft Wearable Electrochemiluminescence Chip for Sweat Metabolite Detection
Source: Adv Sci (Weinh). 2026 Jan 4;13(10):e19435. doi: 10.1002/advs.202519435 (PMC12915171; doi:10.1002/advs.202519435)
Supplement: Supplementary file 1 — Supporting File 1: advs73670‐sup‐0001‐SuppMat.docx. [file ADVS-13-e19435-s002.docx]

**Supporting Information**

**An All-Soft Wearable Electrochemiluminescence Chip for Sweat Metabolite Detection**

*Wei Nie, Jie Jiang,* Xi Wei,* Linfeng Zhuo, Danyang Li, Mingyu Jia, Tianhua Zheng, and Hua Cui**

W. Nie, J. Jiang, X. Wei, L. Zhuo, D. Li, M. Jia, T. Zheng, and H. Cui
Key Laboratory of Precision and Intelligent Chemistry, Department of Chemistry
University of Science and Technology of China
Hefei, Anhui 230026, P. R. China
E-mail: [jiejiang25@ustc.edu.cn,](mailto:jiejiang25@ustc.edu.cn) [wxi@hfut.edu.cn](mailto:wxi@hfut.edu.cn), [hcui@ustc.edu.cn](mailto:hcui@ustc.edu.cn)

X. Wei
Department of Biomedical Engineering, School of Instrument Science and Opto-electronics Engineering
Hefei University of Technology
Hefei, Anhui 230009, P. R. China
E-mail: [wxi@hfut.edu.cn](mailto:wxi@hfut.edu.cn)

**Table of contents**

[S1 Experimental Section S-3](#_Toc216102028)

[S1.1 Chemicals and Materials S-3](#_Toc216102029)

[S1.2 Apparatus S-3](#_Toc216102030)

[S1.3 Configuration of Artificial Sweat S-4](#_Toc216102031)

[S2 Results and Discussion S-4](#_Toc216102032)

[S2.1 Characterization of LIG Electrode S-4](#_Toc216102033)

[S2.2 Design and Optimization of LIG-BPE Array Chip S-7](#_Toc216102034)

[S2.3 Characterization of DES S-8](#_Toc216102035)

[S2.4 Properties of ECL Conductive Ionogel S-9](#_Toc216102036)

[S2.5 ECL Mechanism of the Prepared ECL Conductive Ionogel S-11](#_Toc216102037)

[S2.6 Preparation and Characterization of PEDOT-PB S-13](#_Toc216102038)

[S2.7 Electrochemical Properties of the Prepared PEDOT-PB S-15](#_Toc216102039)

[S2.8 Optimization of Glucose Detection Conditions S-17](#_Toc216102040)

[S2.9 Demonstration of Smartphone Reverse Charging Function S-17](#_Toc216102041)

[S2.10 Stability of the Constructed Glucose Sensing Array Chip S-18](#_Toc216102042)

[S 2.11 Colorimetric Assay S-18](#_Toc216102043)

[S2.12 Response of the Developed ECL Chip to Glucose in Artificial Sweat Samples S-20](#_Toc216102044)

[S2.13 Concentration and Recovery of Glucose in Sweat Samples S-20](#_Toc216102045)

[S2.14 Detailed Dimensions of the Wearable Microfluidic Glucose Sensing Chip S-20](#_Toc216102046)

[S2.15 Physical Pictures of the Microfluidic Cell Filled with Dye S-21](#_Toc216102047)

[S2.16 Commercial Kit for Glucose Determination S-21](#_Toc216102048)

[S2.17 Costing of One Single Wearable ECL Array Chip S-22](#_Toc216102049)

[S 2.18 LOD Calculation S-22](#_Toc216102050)

[References S-23](#_Toc216102051)

# S1 Experimental Section

## S1.1 Chemicals and Materials

Tris(2,2′-bipyridine) dichlororuthenium (II) hexahydrate (Ru(bpy)_3_Cl_2_·6H_2_O) was sourced from TCI (Japan). Glucose, hydrogen peroxide (H_2_O_2_), KCl, NaCl, NH_4_Cl, NaOH, glacial acetic acid, lactate, ascorbic acid (AA), urea, uric acid (UA), potassium ferrocyanide (K_4_Fe(CN)_6_), potassium ferricyanide (K_3_Fe(CN)_6_), iron chloride hexahydrate (FeCl_3_·6H_2_O), ethylene glycol (EG), and choline chloride (ChCl) were obtained from Sinopharm Chemical Reagent Co. Ltd. (Shanghai, China). Phosphate-buffered saline (PBS, 0.1 M), horseradish peroxidase (HRP), and glucose oxidase (GOx) were purchased from Sangon Biotech Co. Ltd. (Shanghai, China). 3,4-ethylenedioxythiophene (EDOT) was acquired from Macklin Biochemical Co. Ltd. (Shanghai, China). Chitosan (CS), N,N-Dibutylethanolamine (DBAE), Diammonium 2,2′-azino-bis(3-ethylbenzothiazoline-6-sulfonate) (ABTS), and tripropylamine (TPA) were supplied by Aladdin Reagent Co. Ltd. (Shanghai, China). Gelatin from porcine skin was obtained from Sigma-Aldrich. All reagents used were of analytical grade. Screen-printed electrodes were purchased from Poten Technology Co., Ltd. (Weihai, China). Kapton polyimide (PI) tape, USB-OTG converter, DC-DC voltage adapter, 3M double-sided tape, and polyethylene terephthalate (PET) plates were purchased online. Indium tin oxide (ITO) electrodes were sourced from Zhuhai Kaivo Optoelectronic Technology Co., Ltd. (Zhuhai, China). Ultrapure water (18.2 MΩ·cm at 25°C) was prepared using a Milli-Q system.

## S1.2 Apparatus

A Laser Engraving Cutting Machine (MIRA5, AEON) was used for preparing laser-induced graphene (LIG) and cutting polyethylene terephthalate (PET) substrates. Scanning electron microscopy (SEM) images were obtained with a GeminiSEM 500 Scanning Electron Microscope (ZEISS, Germany), while transmission electron microscopy (TEM) images were acquired using an HT7700 Exalens Transmission Electron Microscope (Hitachi, Japan). X-ray photoelectron spectroscopy (XPS) was performed using a Thermo ESCALAB 250Xi Electron Spectrograph (VG Scientific, UK) with monochromatic Al Kα radiation. Raman spectra were recorded using a LabRAM Soleil Raman Microscope (HORIBA, Japan). Fourier transform infrared (FT-IR) spectra were measured with a Nicolet iS20 FTIR Spectrometer (Thermo Scientific, USA), and X-ray diffraction (XRD) patterns were collected using a Rigaku Miniflex-600 X-Ray Diffractometer (Japan). Electrochemical tests were conducted with a CHI 760e Electrochemical Workstation (Chenhua, China). A MakerBot Replicator+ 3D Printer (MakerBot, USA) was employed to construct dark boxes. ECL images were captured using a HUAWEI P30 smartphone connected via Bluetooth. Plasma treatments were carried out with a PTL-VM500 Vacuum Plasma Treatment System (PTL Electrical Technology Co., Ltd., China).

## S1.3 Configuration of Artificial Sweat

Artificial sweat (ISO 3160-2) was prepared as follows: 10 g NaCl, 8.75 g NH_4_Cl, 2.5 g urea, 1.25 g acetic acid and 7.5 g lactic acid were added to 450 mL of ultrapure water, the pH was adjusted to 5.5 with 2 M NaOH, and the final volume was fixed to 500 mL with ultrapure water.

# S2 Results and Discussion

## S2.1 **Characterization of LIG Electrode**

First, the output power of the laser engraving machine was optimized by measuring the resistance of a specified length of LIG (Figure S1). The LIG prepared at the optimized power (5.4 W) was characterized through a series of methods. The morphology and microstructure of LIG were observed by SEM images. As shown in Figure S2A, the LIG engraved directly on the PI film shows a continuous groove structure. On the magnified LIG image of Figure S2A, the connected porous structure and the thin layer of graphene can be clearly seen, which was due to the high-temperature carbonization of the PI film. These porous structures of LIG enhance the surface area, accelerate electron transfer, and provide abundant active sites.^[1]^ The Raman spectrum of LIG (Figure S2B) shows three characteristic bands: the D band at 1346 cm^-1^, the G band at 1581 cm^-1^, and the 2D band at 2700 cm^-1^. D band refers to constructional defects of graphene, G band suggests the E_2g_ vibration nature of graphitic carbon, and 2D band is dependent on the layers of graphene. The D and G bands correspond exactly to the typical peaks of graphene materials. Additionally, the intensity ratio of the D and G bands (I_D_/I_G_) was 0.61, revealing a highly defective graphene structure, while a low value of I_2D_/I_G_ (0.63) indicated the existence of multiple-layer graphene.^[2]^ The crystal structure of LIG was then tested by XRD. As seen in Figure 2C, the XRD pattern shows two distinct peaks at 2θ = 26.0° and 2θ = 42.48°, which correspond to the (002) and (100) planes of graphene, respectively.^[3]^


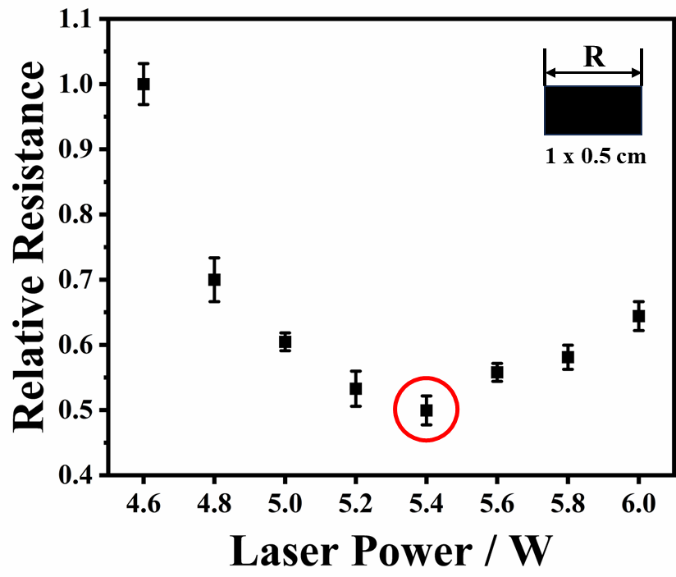


**Figure S1.** Relative resistance of 1×0.5 cm shaped rectangular patterns at different laser output powers.

Additionally, XPS analysis was performed to further investigate the composition and chemical states of various elements in the prepared LIG. As shown in Figure S2D, the full XPS spectrum indicated that the LIG contained mainly two elements, C and O, in which carbon dominated, suggesting that the PI film was highly carbonized after laser irradiation. Figure S2E shows the deconvolution XPS spectrum of C 1s in LIG, where three peaks located at 284.3, 285.4 and 288.5 eV can be assigned to C-C/C=C, C-O and C=O, respectively. In addition, O1s (Figure S2F) can be assigned to C-O (532.7 eV) and C=O (531.8 eV), and the C-O peak was more dominant than the C=O peak, which was consistent with previously reported literature.^[2,4]^ All the above results indicated the successful preparation of porous LIG.


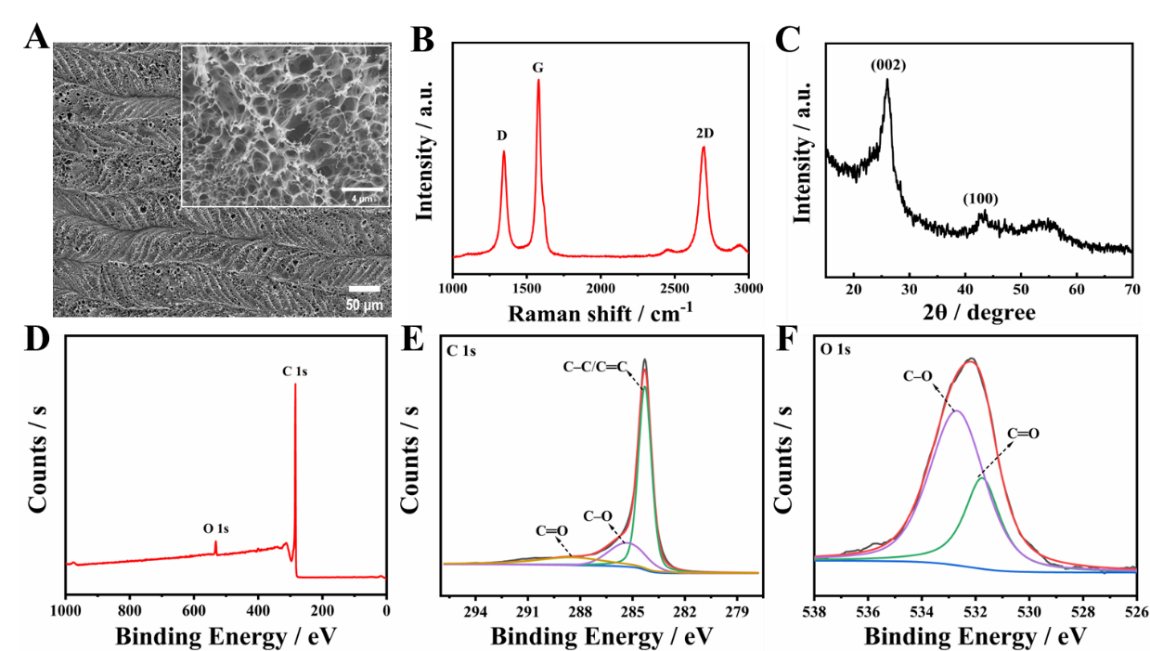


**Figure S2.** (A) SEM image of LIG. Inset: high magnification SEM image. (B) Raman spectrum of LIG. (C) XRD pattern of LIG. (D) Full XPS spectrum of LIG. Deconvolution XPS spectra of C 1s (E) and O 1s (F) of LIG.

## S2.2 Design and Optimization of LIG-BPE Array Chip


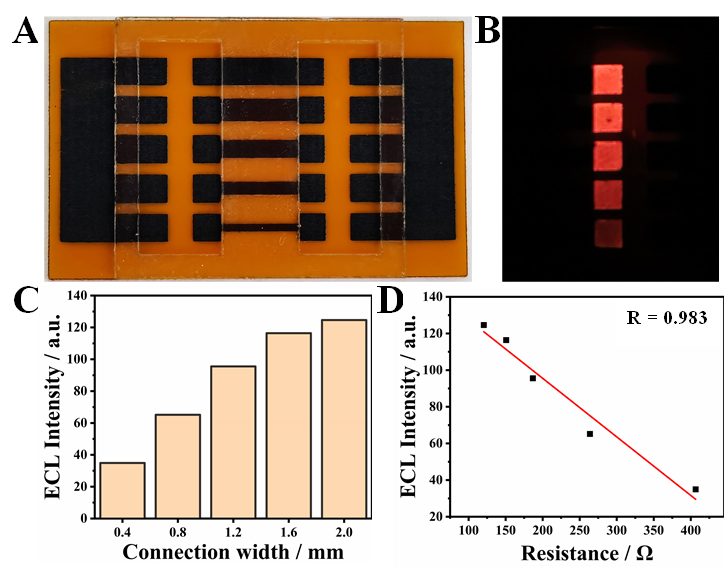


**Figure S3.** Physical (A) and ECL images (B) of the array chip with BPE electrodes of different resistance values. (C) ECL intensities of different BPE connection section widths. (D) BPE connection section resistance-ECL intensity curve (E_tot_ = 3.8V).


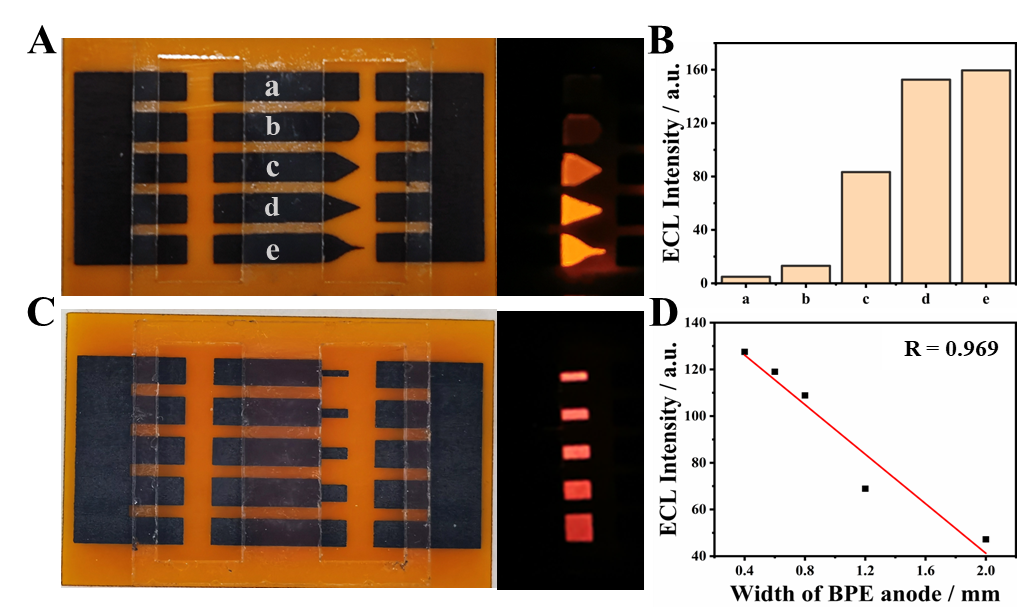


**Figure S4.** (A) Physical and ECL images of BPE array chip with different anode shapes. From top to bottom, the anode top angles are 90°, 60°, 40°, and 15°. (B) ECL intensities correspond to BPE with different anode shapes. (C) Physical and ECL images of BPE array chip with different anode widths. (D) BPE anode width-ECL intensity curve (E_tot_ = 3.0 V). The length dimensions at the bottom of all anodes were kept constant at 2 mm.


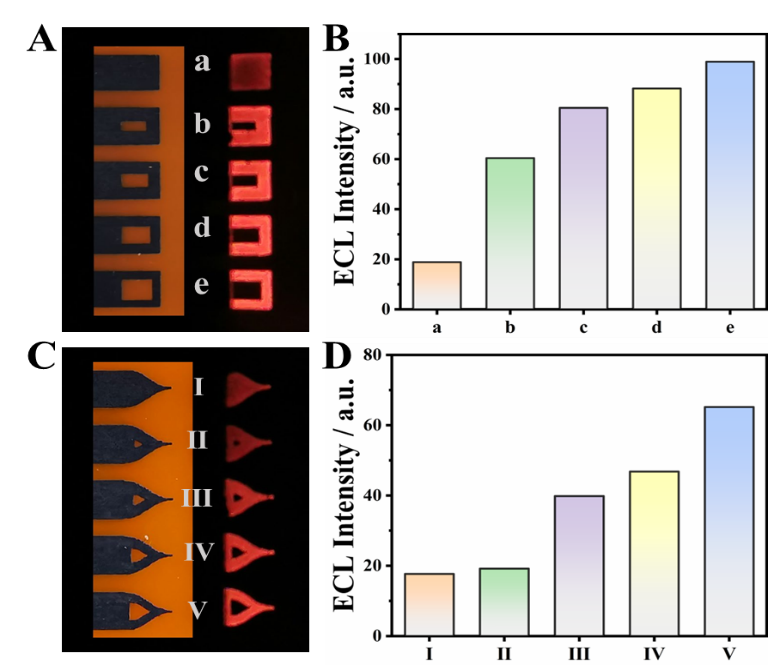


**Figure S5.** Physical and ECL images (A) and comparison of corresponding ECL intensities (B) of rectangular-shaped BPE with different skeletonization ratios (E_tot_ = 3.2V). Physical and ECL images (C) and corresponding ECL intensity comparisons (D) of writing brush-shaped BPE with different skeletonization ratios (E_tot_ = 3.0V).

## S2.3 Characterization of DES

The preparation of DES was characterized to confirm its formation. The chemical structural formulas of ChCl, EG, and urea are shown in Figure S6A. DES was prepared by mixing these components in a molar ratio of 1:2:1. As illustrated in Figure S6B, ChCl served as the sole hydrogen bond acceptor, forming hydrogen bonds via its Cl atom with the hydroxyl groups of EG (-OH) and the amino groups of urea (-NH₂).^[5]^ The results were validated using FT-IR spectroscopy. As shown in Figure S6C, peaks at 3210 cm^-1^ and 3300 cm^-1^ were attributed to the -OH groups of ChCl and EG, respectively, while urea exhibited two peaks at 3330 cm^-1^ and 3430 cm^-1^, corresponding to the symmetric and antisymmetric stretching vibrations of -NH₂. Additionally, a peak at 1675 cm^-1^, representing the -C=O group in urea, was also observed in the DES spectrum. The DES spectrum also showed a series of absorption peaks in the range 800−1150 cm^-1^, resulting from the superposition of peaks from ChCl and EG. Most significantly, the peaks corresponding to the individual components in the 3200−3450 cm^-1^ range (ChCl: 3210 cm^-1^, EG: 3300 cm^-1^, urea: 3330 and 3430 cm^-1^) merged to form a strong, broad absorption peak at 3320 cm^-1^, confirming the formation of hydrogen bonds.^[6]^ These results collectively indicate the successful synthesis of DES.


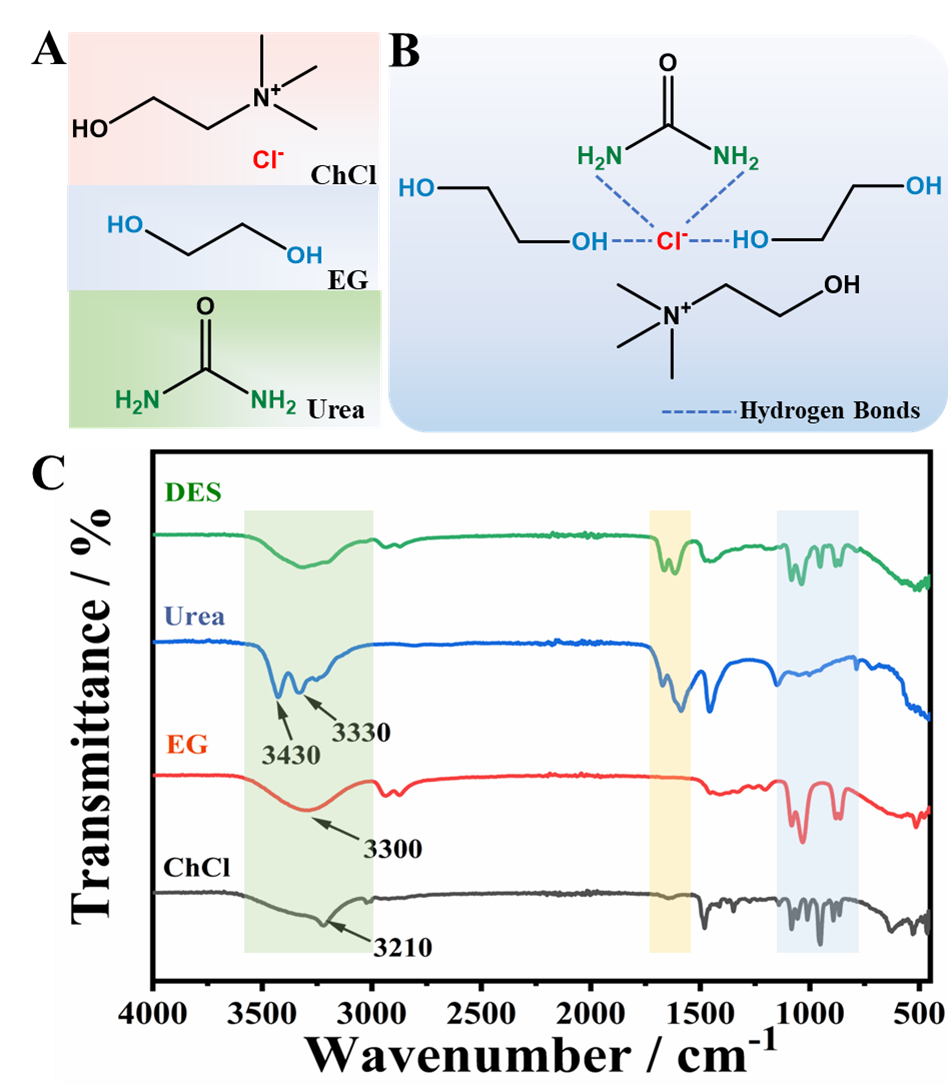


**Figure S6.** (A) Chemical structural formulas of ChCl, EG and urea. (B) Hydrogen bonds between EG, urea, and ChCl. (C) FT-IR spectra of ChCl, EG, urea, and DES.

## S2.4 Properties of ECL Conductive Ionogel


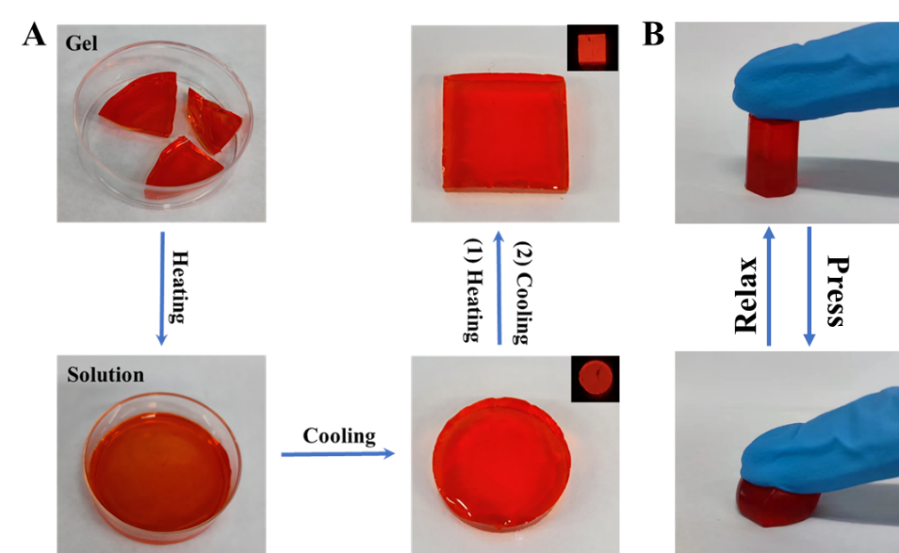


**Figure S7.** Reprocessing properties (A) and elasticity (B) of the synthesized ECL conductive ionogel. Inset: ECL images of ECL gels with different shapes.

**
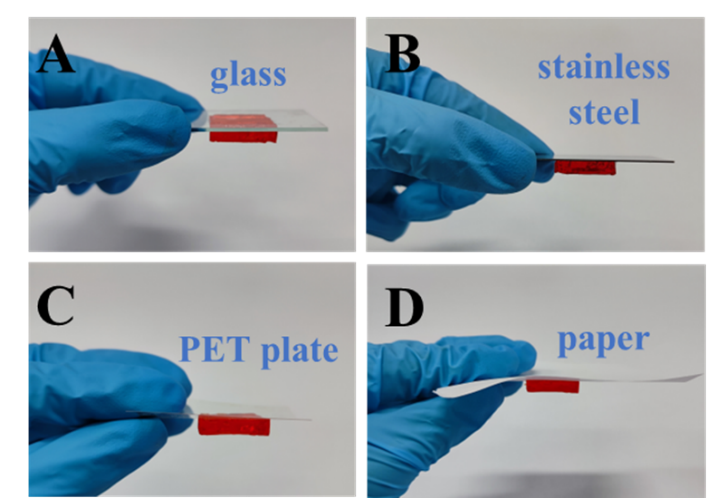
**

**Figure S8.** Photographs of ECL gel adhered to glass (A), stainless steel (B), PET plate (C), and paper (D).

In order to determine the ionic conductivity of the ECL ionogel, the gel was sandwiched between two stainless steel electrodes and AC impedance tests were performed between 10^-1^ Hz and 10^5^ Hz. The ionic conductivity (σ) can be calculated from the equation σ = L / (S × R_s_), where L is the thickness of the gel, S represents the contact area between the electrodes and the ionogel, and R_s_ is the resistance of the ionogel derived from the intercept of the real axis in the AC impedance curve.

**
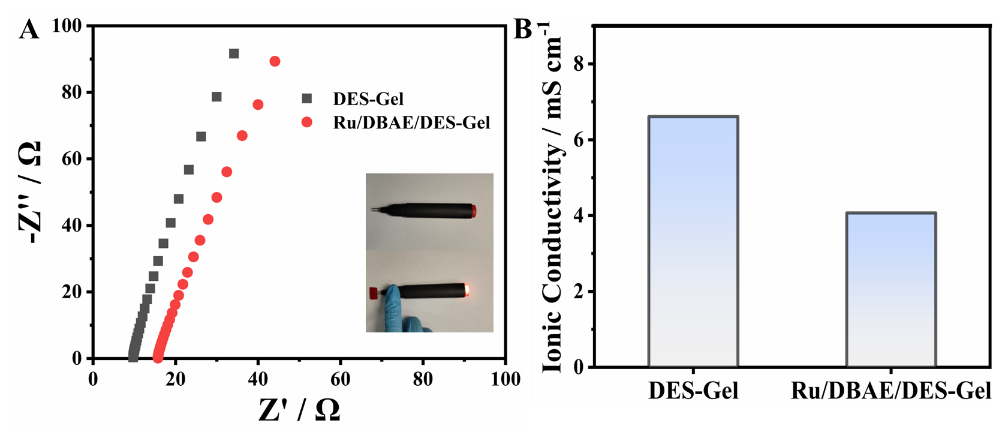
**

**Figure S9.** Electrochemical impedance spectroscopy of DES-Gel and Ru/DBAE/DES-Gel. Inset: Changes in the conductive pen before (up) and after (down) contact with ECL conductive ionogel. (B) Ionic conductivities of DES-Gel and Ru/DBAE/DES-Gel.





**Figure S10.** ECL intensities of the prepared ECL display device under different DC voltages.

## S2.5 ECL Mechanism of the Prepared ECL Conductive Ionogel

First, screen-printed electrodes (with Ag/AgCl as the reference electrode) were utilized to investigate the ECL behavior of Ru(bpy)_3_^2+^/DBAE in liquid-phase DES. As shown in Figure S11B, no ECL signal was observed in either the DES or Ru/DES systems. Conversely, the Ru/DBAE/DES system exhibited a strong ECL signal, with a peak potential near +1.3 V under a cyclic voltammetry (CV) scan of 0−1.6 V. This result indicates that DES itself does not function as an ECL co-reactant, serving primarily as a non-volatile conductive matrix within the gel network, whereas DBAE plays a crucial role as a co-reactant in enhancing the ECL reaction. This result highlights the critical role of DBAE as a co-reactant in enhancing the ECL response. Further investigation of the ECL emission mechanism was conducted using CV curves (Figure S11A). A new oxidation peak near +0.9 V in the Ru/DBAE/DES system, absent in systems without DBAE, was attributed to the electrooxidation of DBAE. Additionally, the CV onset potential for Ru/DES was observed at +1.2 V, corresponding to the onset of ECL emission, indicating that Ru(bpy)_3_^2+^ begins to electrooxidize at this potential. The ECL intensity of the Ru/DBAE/DES system remained stable after multiple pulse voltage applications (Figure S11C), demonstrating the excellent stability of Ru(bpy)_3_^2+^ in this system.

The ECL mechanism of the prepared ECL conductive ionogel was further investigated. Due to the excellent conductivity of the prepared ECL gel, it can be tested directly on screen-printed electrodes without the need for additional buffer solutions. As shown in Figure S11E, Ru/DES-Gel exhibited a weak ECL response, likely due to amino groups in the gelatin structure acting as co-reactants for the Ru(bpy)_3_^2+^ ECL reaction. Upon the addition of DBAE, the ECL intensity significantly increased, with a starting potential of approximately +1.1 V and a peak potential near +1.3 V, consistent with the ECL emission observed in the Ru(bpy)_3_^2+^/DBAE system in liquid-phase DES. Furthermore, CV curves for the conducting gels (Figure S11D) demonstrated that the gel state did not affect the ECL behavior of the Ru(bpy)_3_^2+^/DBAE system. The ECL emission wavelength of the prepared conductive gel during CV scanning was recorded using 3D ECL spectroscopy. The gel exhibited ECL emission with a starting potential of approximately +1.1 V, peaking at +1.3 V, and a wavelength of about 619 nm, attributed to Ru(bpy)_3_^2+^ emission (Figure S11F). Based on these findings and prior studies, the ECL emission mechanism of Ru(bpy)_3_^2+^/DBAE in the conductive gel, with DES as the electrolyte and gelatin as the polymer backbone, is summarized in equations (S1) to (S5). Briefly, DBAE undergoes electrooxidation to form radical cations, which rapidly deprotonate to generate DBAE radicals. Simultaneously, Ru(bpy)_3_^2+^ is electrochemically oxidized to Ru(bpy)_3_^3+^. Intramolecular electron transfer then produces the excited state (Ru(bpy)_3_^2+^*), which decays to the ground state with ECL emission.^[7]^

DBAE – e^−^ → DBAE^+•^ (S1)

DBAE^+•^ → DBAE^•^ + H^+^ (S2)

Ru(bpy)_3_^2+^ – e^−^ → Ru(bpy)_3_^3+^ (S3)

DBAE^•^ + Ru(bpy)_3_^3+^ → Ru(bpy)_3_^2+^* (S4)

Ru(bpy)_3_^2+^* → Ru(bpy)_3_^2+^ + *hv* (S5)


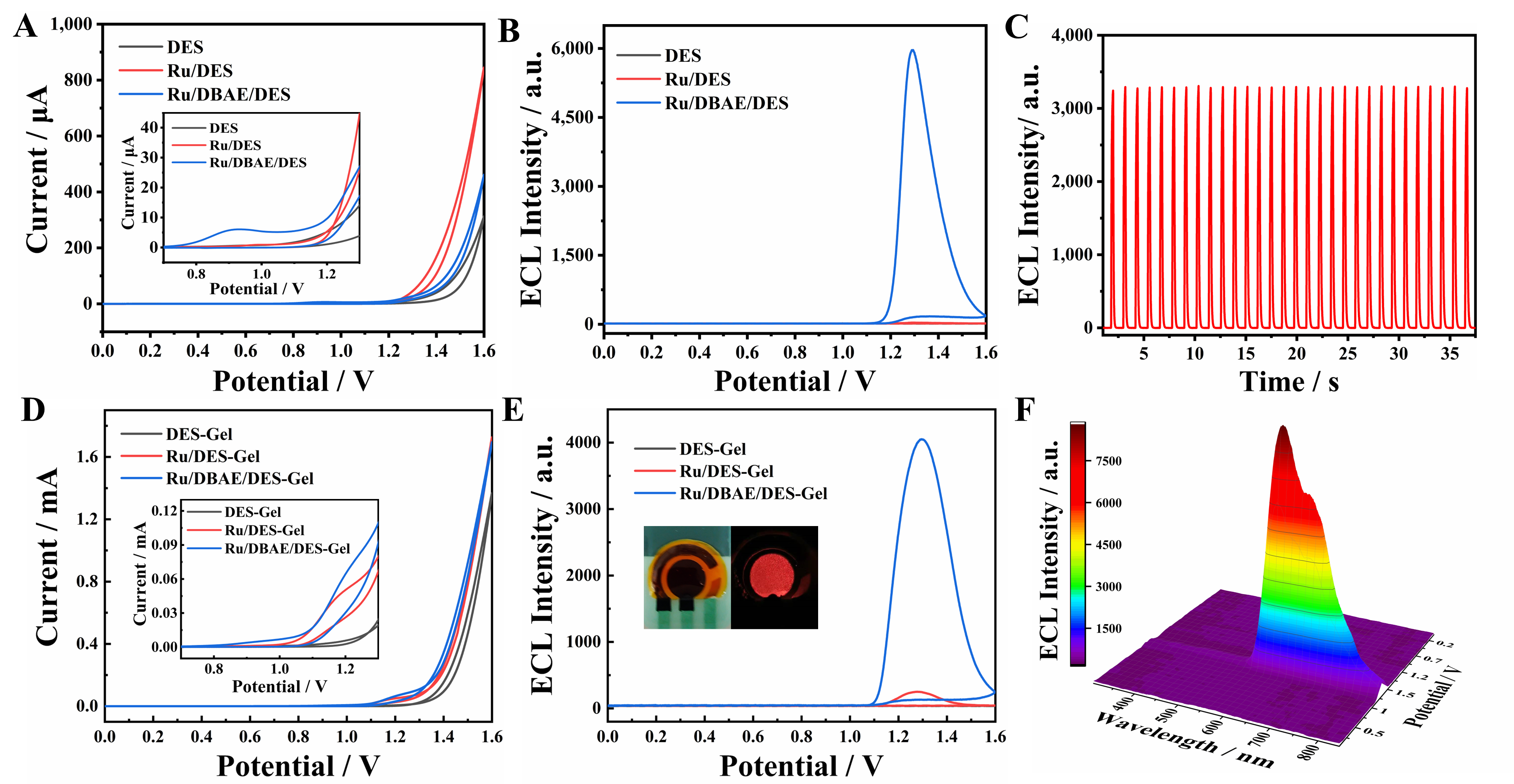


**Figure S11**. CV curves (A) and ECL-potential curves (B) of DES (black curve), Ru/DES (red curve), and Ru/DBAE/DES (blue curve). (C) ECL signals of Ru/DBAE/DES under pulse potential. Initial potential 0 V; pulse time 0.5 s; pulse period 1.0 s; pulse potential 1.3 V. CV curves (D) and ECL-potential curves (E) of DES-Gel (black curve), Ru/DES-Gel (red curve), and Ru/DBAE/DES-Gel (blue curve). Inset: Physical and ECL emission image of ECL gel-modified screen-printed electrode. (F) 3D ECL spectra of the prepared ECL ionogel.

## S2.6 Preparation and Characterization of PEDOT-PB

PEDOT-PB was obtained by mixing the two precursor solutions.^[8]^ Briefly, 10 mL of a 0.1 M HCl aqueous solution containing 4 mM FeCl_3_ and 4 mM K_3_Fe(CN)_6_ was sonicated for 10 min to form solution A. At the same time, 5 mL of ethanol containing 50 μL of EDOT was added to 25 mL of the 0.1 M HCl solution to form solution B. Under vigorous agitation, solution A was slowly added to solution B. The resulting suspension was stirred overnight at room temperature. The mixture gradually changed from green color to dark blue color, indicating the formation of PB-PEDOT nanocomposites. Finally, the mixture was centrifuged and washed repeatedly with ethanol and water and dried at 40°C. The final product obtained was stored in a sealed container. During the synthesis process, the generation of PB occured simultaneously with the polymerization of EDOT. EDOT was not only a precursor of PEDOT, but also acted as a weak reducing agent in the conversion of Fe^3+^ to Fe^2+^. The core-shell structure of PB-PEDOT improved the stability and electrical conductivity of PB.

The synthesized PEDOT-PB nanocomposites were characterized by TEM, SEM and FT-IR spectroscopy. Figure S12A shows the TEM image of the PB-PEDOT core-shell nanocomposites. It can be seen that the PB nanoparticles were completely encapsulated by PEDOT, which can effectively improve the stability and conductivity of PB.^[9]^ In addition, the SEM image of PB-PEDOT presented a three-dimensional multistage porous structure (Figure S12B), which not only provided a favorable microenvironment and large specific surface area, but also promoted direct electron transfer. From the energy dispersive X-ray spectrum (EDS) and elemental mapping of PEDOT-PB, it can be seen that the main constituent elements of PEDOT-PB include C, N, O, Fe, and S (Figure S12C and Fig S12D). Figure S13 shows the FT-IR spectra of PB and PEDOT-PB. The absorption peak at 2070 cm^-1^ shows a common feature of PB and its analogs, corresponding to the stretching vibration of the CN group.^[10]^ The absorption peak at 491 cm^-1^ may be related to the Fe-CN-Fe bending mode, which indicated the presence of PB.^[8]^ Moreover, in the FT-IR spectrum of PEDOT-PB, the absorption peaks located at 1519 cm^-1^ and 1355 cm^-1^ were caused by the C-C and C=C stretching of the quinone structure of the thiophene ring in the synthesized PEDOT.^[11]^ The absorption peaks located at 1210 cm^-1^ and 1143 cm^-1^ correspond to the stretching vibration of the C-O-C of the ethylene dioxygen group, while the absorption peaks at 1089 cm^-1^ and 1055 cm^-1^ correspond to its bending vibration. In addition, the absorption peaks located at 981 cm^-1^, 838 cm^-1^, and 691 cm^-1^ could be categorized as C-S-C stretching vibrations.^[11]^ All of the above results indicated that the successful synthesis of PEDOT-PB.


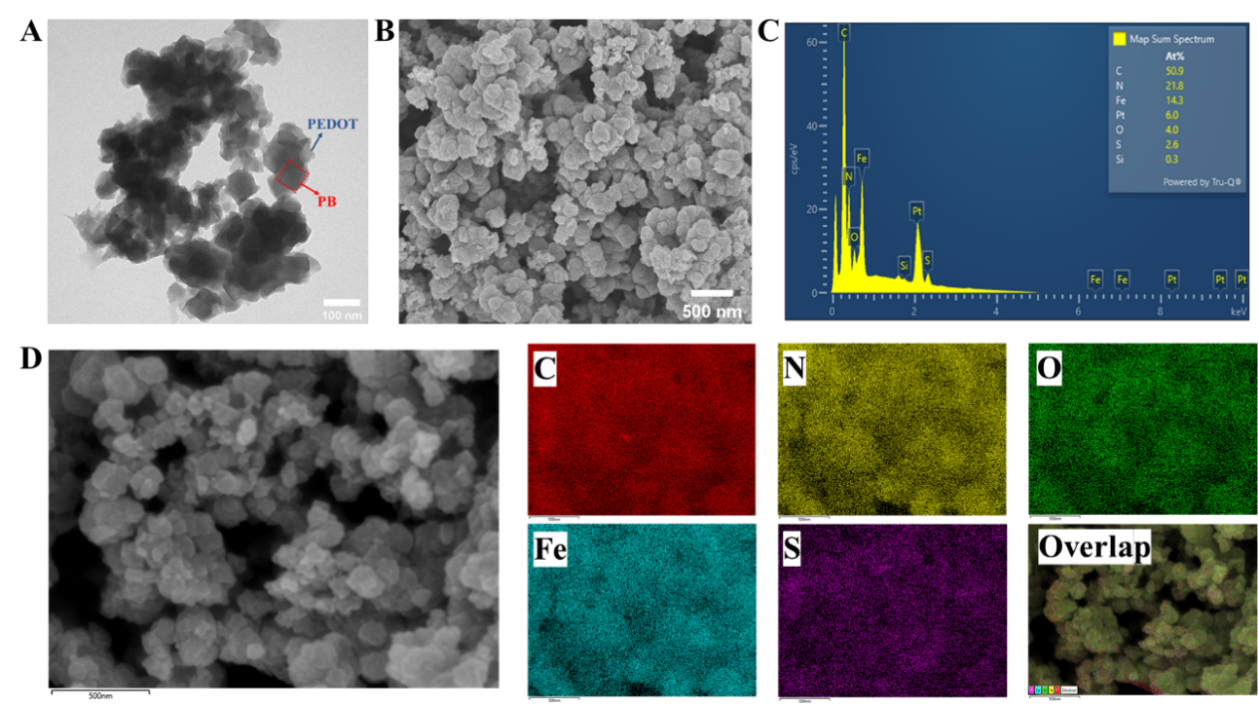


**Figure S12.** TEM (A) and SEM (B) images of PEDOT-PB. (C) The EDS spectrum of PEDOT-PB. (D) SEM image and elemental mapping analysis of PEDOT-PB.


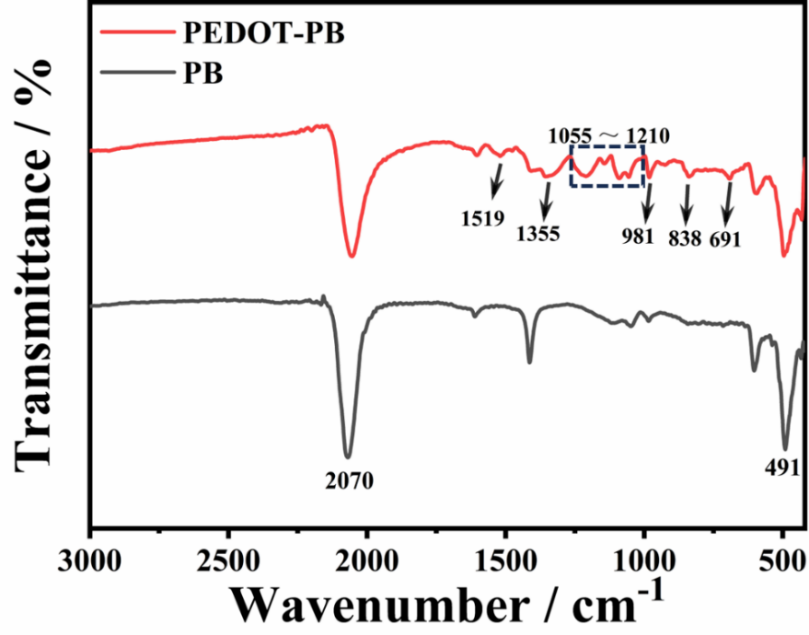


**Figure S13.** FT-IR spectra of PB and PEDOT-PB.

## S2.7 Electrochemical Properties of the Prepared PEDOT-PB

The electrochemical performance of PEDOT-PB was investigated by means of a conventional three-electrode system, in which the PEDOT-PB-modified glassy carbon electrode (GCE) was used as the working electrode, the Ag/AgCl electrode as the reference electrode, and the Pt electrode as the counter electrode. As shown in Figure S14A, the CV curves of both PB and PEDOT-PB-modified GCE in 0.1 M KCl showed a pair of redox peaks, where the cathodic and anodic peaks refered to electrochemically-induced reduction of PB to Prussian white (PW) in the forward scan and oxidation of PW to PB in the reverse scan, respectively. However, the current corresponding to PEDOT-PB was significantly larger than that of PB, which was attributed to the good electrical conductivity and redox activity of PEDOT-PB. The electrochemical impedance spectroscopy (EIS) further verified that the conductivity of PEDOT-PB was superior to that of PB (Figure S14B), which was closely related to the good conductivity of PEDOT. Both the oxidation and reduction currents of PEDOT-PB became progressively larger as the scan rate increased (Figure S14C), and there was a good linear relationship between the peak current of the PB-PW redox pair and the square root scan rate (Figure S14D). This phenomenon suggested that this was a diffusion-controlled process facilitated by the movement of potassium ions through the PB, indicating the suitability of the PB for use in quantitative sensors. The anodic and cathodic peak potentials shifted outward as the scan rate increased, indicating quasi-reversible behavior.^[12]^


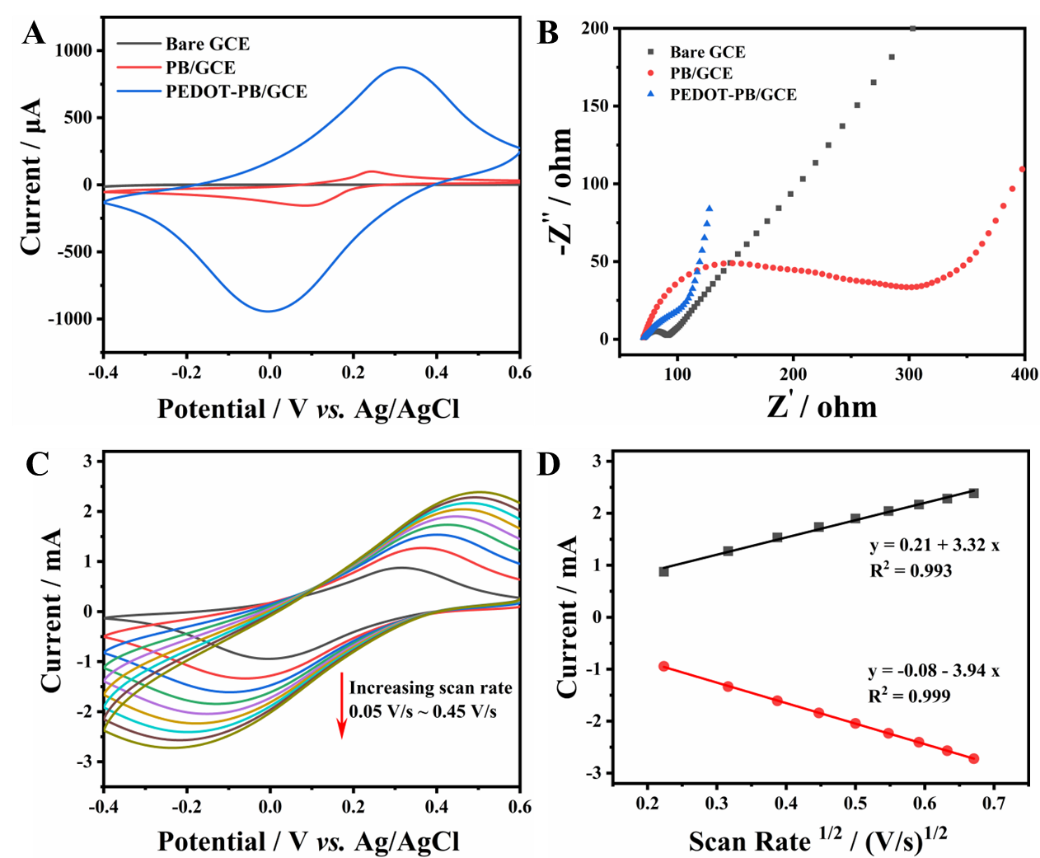


**Figure S14.** (A) CV curves of bare GCE (black curve), PB/GCE (red curve), and PEDOT-PB/GCE (blue curve) in 0.1 M KCl solution. (B) EIS curves of bare GCE (black curve), PB/GCE (red curve), and PEDOT-PB/GCE (blue curve) in 5 mM Fe(CN)_6_^3-/4-^/0.1 M KCl solution. (C) CV curves of PEOT-PB modified GCE under different scan rates. (D) Linear relation between the peak currents of PEOT-PB modified GCE and the square root of scan rate.

## S2.8 Optimization of Glucose Detection Conditions

The optimal conditions for the glucose detection were optimized, including the concentration of modified PEDOT-PB, the magnitude of the applied potential, and the enzymatic reaction time. As shown in Figure S14A, the ECL intensity was first enhanced with the increase of the concentration of PEDOT-PB and stabilized at 3 mg/mL, so 3 mg/mL was chosen as the optimal value of PEDOT-PB concentration. For the applied potential, the ECL intensity was approximately “S” shaped with respect to the magnitude of the applied potential (Figure S14B), and an optimized potentialof 2.6 V was chosen in order to obtain a large ECL intensity at as low a potential as possible. In this regard, a lower potential can avoid the occurrence of some side reactions. The enzymatic reaction time catalyzed by GOx after the buffer containing glucose was added to the chip sensing zone was also optimized, as shown in Figure S14C, the ECL intensity remained essentially unchanged after a reaction time of 5 min, and thus 5 min was chosen as the optimal enzymatic reaction time.


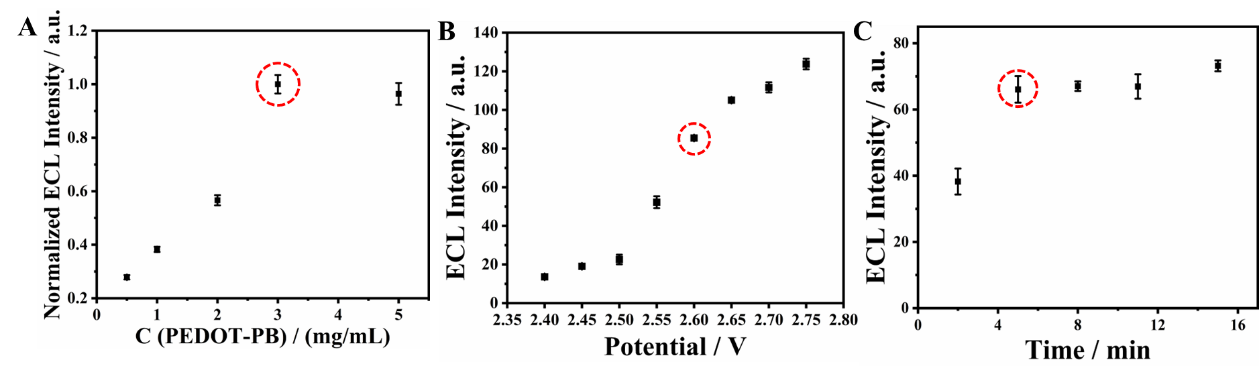


**Figure S15.** Effect of PEDOT-PB concentration (A), applied potential (B), and enzymatic reaction time (C) on the ECL intensity of the constructed array chip.

## S2.9 Demonstration of Smartphone Reverse Charging Function

The smartphone was reverse-charged via USB-On The Go (USB-OTG), and the magnitude of the output voltage could be adjusted by a DC-DC voltage adapter. (Figure S16A). In addition, it can be seen that the ECL intensities of the chip were essentially the same when used as a power source from smartphones and DC power supplies (Figure S16B), verifying the reliability of the smartphone as a power output.


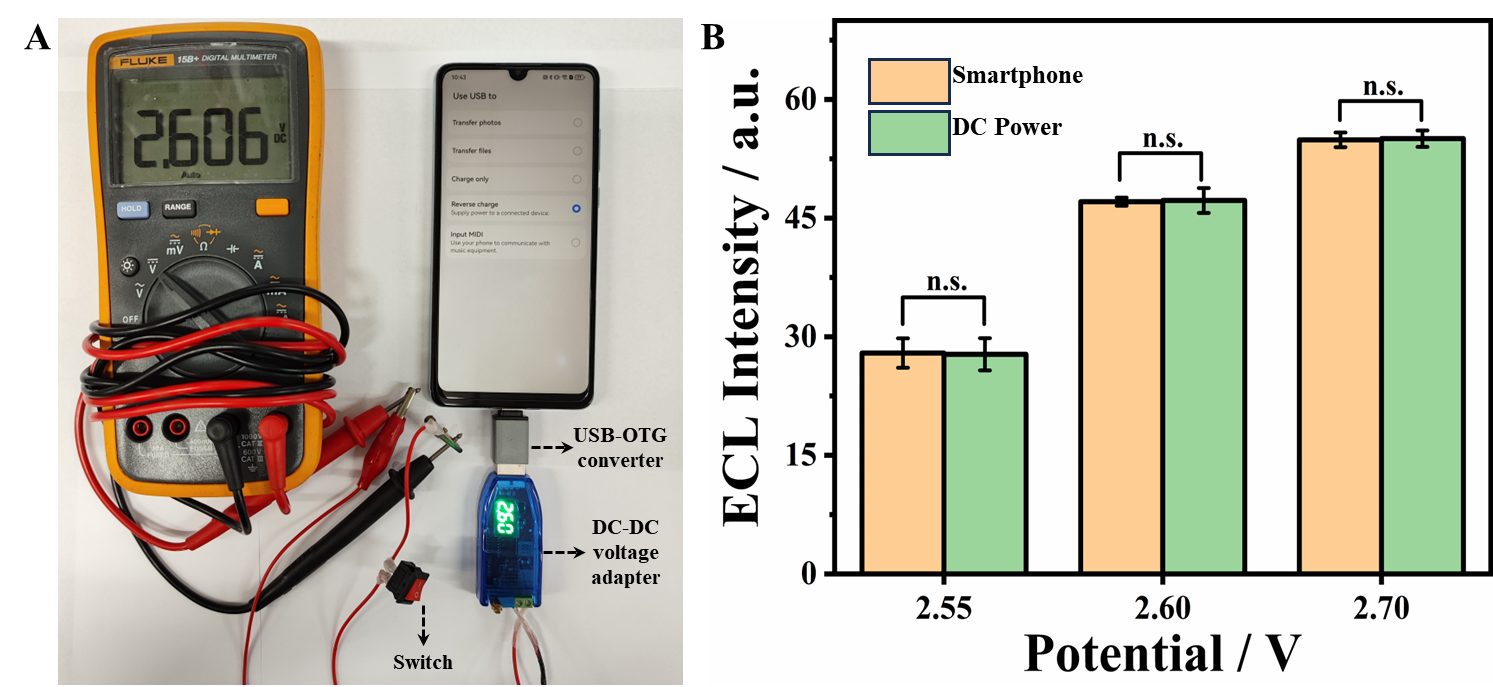


**Figure S16.** (A) Picture of the smartphone outputting 2.6V through the reverse charging function. (B) ECL intensities of the chip corresponding to the smartphone power supply and the DC power supply. n.s., not significant.

## S2.10 Stability of the Constructed Glucose Sensing Array Chip


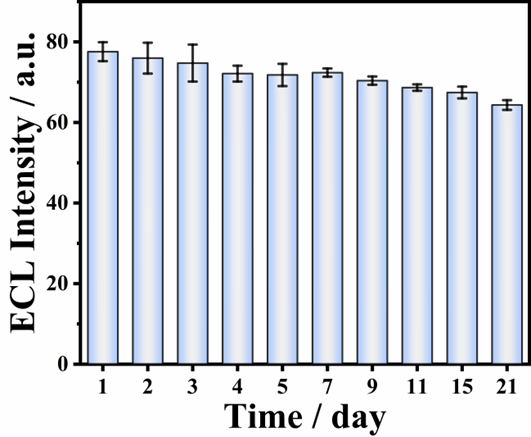


**Figure S17.** Stability of the constructed glucose sensing array chip (C_glucose_ = 0.5 mM).

## S 2.11 Colorimetric Assay

To verify the activity of GOx modification in the sensing zone, the post-reaction solution underwent a colorimetric assay using the Diammonium 2,2’-azino-bis(3-ethylbenzothiazoline-6-sulfonate) (ABTS)/horseradish peroxidase (HRP) mixture. This colorimetric reaction (ABTS/HRP + H_2_O_2_) is a well-established and widely accepted method for detecting H_2_O_2_ in biochemistry.^[13,14]^ In the presence of H_2_O_2_—the enzymatic product of glucose oxidation by GOx—HRP catalyzes the oxidation of colorless ABTS to a stable green-colored radical cation (ABTS^•^⁺) (Figure S18A). We conducted colorimetric control experiments by reacting different test solutions (20 µL) with the ABTS/HRP mixture (80 µL) for 10 min. The results demonstrated that only the solution resulting from glucose reaction in the chip’s reporting zone and the H_2_O_2_ solution can cause the ABTS/HRP mixture to turn green (Figure S18B), confirming that glucose successfully generates H_2_O_2_ through enzymatic reaction in the reporting zone. Furthermore, we have further confirmed the formation of green oxidation product ABTS^•^⁺ via UV-vis spectroscopy, which exhibits characteristic absorption peaks at 416 nm, 646 nm, 728 nm, and 821 nm,^[15]^ as shown in Figure S18C. The aforementioned results confirmed the high catalytic activity of GOx modified in the sensing zone for glucose oxidation and the generation of substantial amounts of H_2_O_2_.


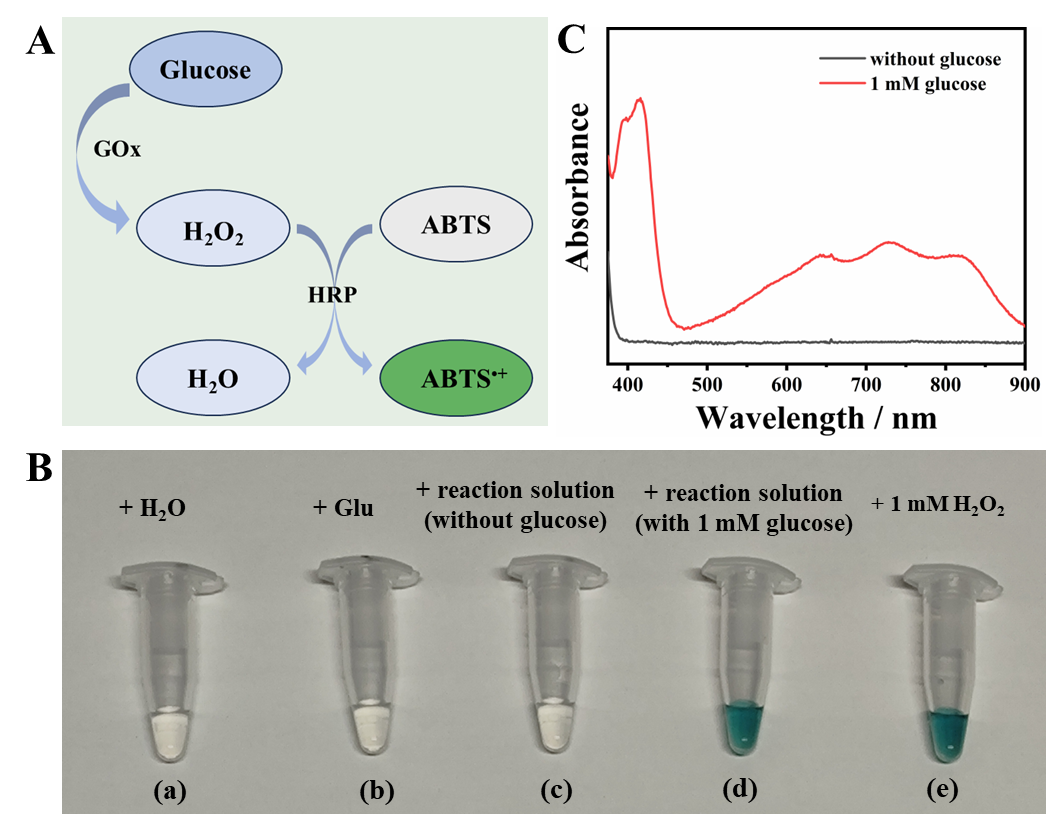


**Figure S18.** (A) Schematic diagram of the principle of colorimetric assay. (B) Photographs showing color development after 10 min of reaction between different test solutions and the ABTS (50 μM)/HRP (0.1 μM) mixture. Test solution categories: (a) H2O, (b) 1 mM glucose, (c) solution after reaction in the sensing zone (without glucose), (d) solution after reaction in the sensing zone (1 mM glucose), (e) 1 mM H2O2. (C) UV-vis spectra of the solution after reaction in the sensing zone (with/without glucose) and the ABTS/HRP mixture.

## S2.12 Response of the Developed ECL Chip to Glucose in Artificial Sweat Samples





**Figure S19.** Relationship curve between the ECL intensity of the developed ECL chip and the logarithm of the glucose concentration in artificial sweat samples.

## S2.13 Concentration and Recovery of Glucose in Sweat Samples

| Sweat Sample | Initial  (μM) | Added  (μM) | Found  (μM) | Recovery (%) |
| --- | --- | --- | --- | --- |
| 1 | 61.1 ± 1.8 | 50.0 | 110.3 ± 6.9 | 98.4 |
|  |  | 100.0 | 168.9 ± 6.8 | 107.8 |
| 2 | 109.3 ± 5.9 | 50.0 | 156.8 ± 12.7 | 94.9 |
|  |  | 100.0 | 218.4 ± 10.2 | 109.1 |

**Table S1.** Concentration and recovery of glucose in sweat samples.

## S2.14 Detailed Dimensions of the Wearable Microfluidic Glucose Sensing Chip


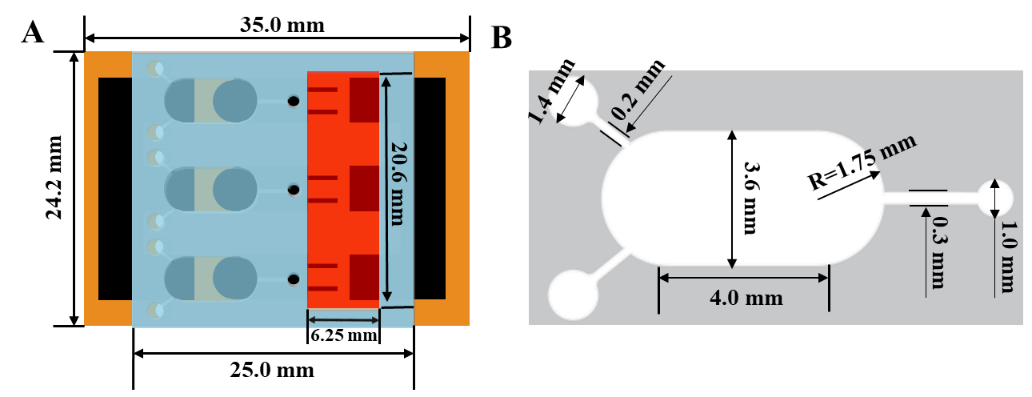


**Figure S20.** Schematic and detailed dimensions of the wearable glucose sensing array chip (A) and microfluidic cell (B).

## S2.15 Physical Pictures of the Microfluidic Cell Filled with Dye


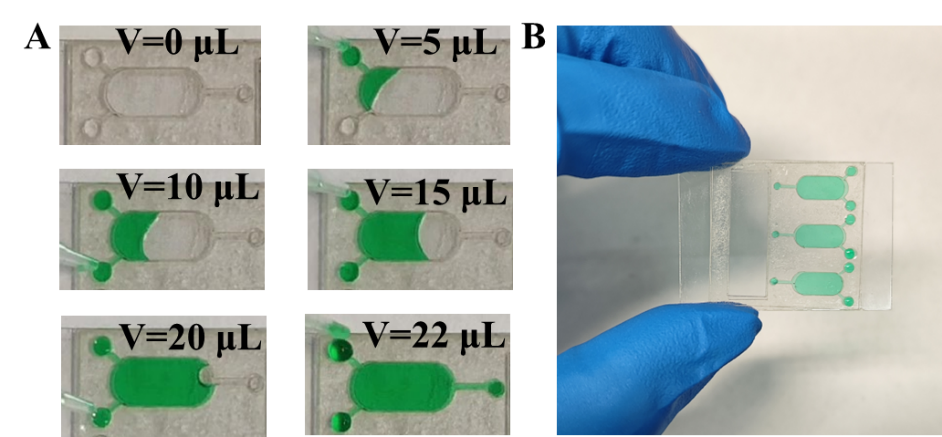


**Figure S21.** (A) Physical pictures of different volumes of dye after addition to the microfluidic cell. (B) Physical picture of the microfluidic cell after it was filled with dye and picked up.

## S2.16 Commercial Kit for Glucose Determination


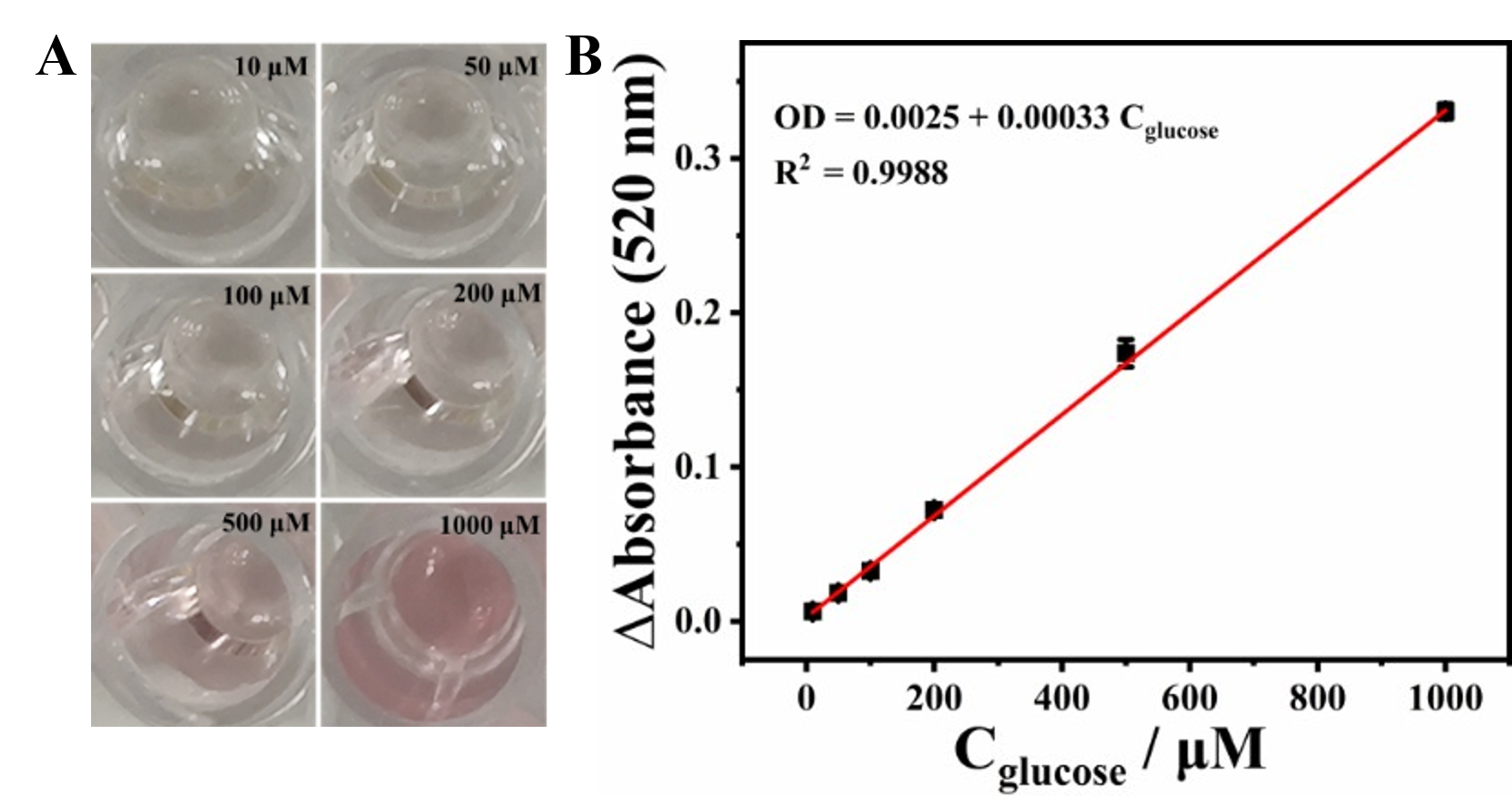


**Figure S22.** (A) The colorimetric responses of a commercial glucose assay kit toward glucose with various concentrations from 10 to 1000 μM. (B) The linear relationship between the absorbance to the glucose concentration.

## S2.17 Costing of One Single Wearable ECL Array Chip

**Table S2.** Estimate cost analysis for fabrication of one chip.

| Materials and chemicals | Amount per chip | Price per unit | Price per chip |
| --- | --- | --- | --- |
| PI tape | 3.5 × 2.42 cm^2^ | ¥0.0065/cm^2^ | ¥0.055 |
| PET plate | 2.5 ×2.42 cm^2^ ×3 | ¥0.0016/cm^2^ | ¥0.029 |
| Double-side tape | 3.5 × 2.42 cm^2^ and  2.5 ×2.42 cm^2^ ×3 | ¥0.0011/cm^2^ | ¥0.029 |
| Glucose oxidase | 0.09 mg | ¥3.95/mg | ¥0.36 |
| Ru(bpy)_3_Cl_2_·6H_2_O | 0.2 mg | ¥0.98/mg | ¥0.196 |
| Other chemicals | − | − | ¥0.10 |
| Total price per chip: ¥0.769 ($0.106） | | | |

## S 2.18 LOD Calculation

The smallest detectable ECL intensity could be calculated as follows: I_LOD_ = b + kS_0_.^[16,17]^ The ECL intensities of blank samples were carried out with seven parallel tests, which performed average ECL intensity·(b) of 25.22 a.u. with·standard·deviation (S_0_) of 0.74. With signal-to-noise ratio value·(k) of 3, the smallest detectable ECL intensity could be calculated as follows: I_LOD_ = 25.22 + kS_0_ = 27.44 (a.u.). According to the linear regression equation I_ECL_ = -8.51+ 31.08*lgC_glucose_, the LOD was calculated as 14.3 μM.

# References

[1] Z. Lu, L. Wu, X. Dai, Y. Wang, M. Sun, C. Zhou, H. Du, H. Rao, Novel flexible bifunctional amperometric biosensor based on laser engraved porous graphene array electrodes: Highly sensitive electrochemical determination of hydrogen peroxide and glucose*.* *J. Hazard. Mater.* **2021**, *402*, 123774.

[2] Y. Wang, Y. Li, C. Liu, N. Dong, D. Liu, T. You, Laser induced graphene electrochemical aptasensor based on tetrahedral DNA for ultrasensitive on-site detection of microcystin-LR*.* *Biosens. Bioelectron.* **2023**, *239*, 115610.

[3] T. Raza, M. K. Tufail, A. Ali, A. Boakye, X. Qi, Y. Ma, A. Ali, L. Qu, M. Tian, Wearable and Flexible Multifunctional Sensor Based on Laser-Induced Graphene for the Sports Monitoring System*.* *ACS Appl. Mater. Interfaces* **2022**, *14*, 54170-54181.

[4] Y. Zhang, N. Li, Y. Xiang, D. Wang, P. Zhang, Y. Wang, S. Lu, R. Xu, J. Zhao, A flexible non-enzymatic glucose sensor based on copper nanoparticles anchored on laser-induced graphene*.* *Carbon* **2020**, *156*, 506-513.

[5] M. Zhong, Q. F. Tang, Z. G. Qiu, W. P. Wang, X. Y. Chen, Z. J. Zhang, A novel electrolyte of ternary deep eutectic solvent for wide temperature region supercapacitor with superior performance*.* *J. Energy Storage* **2020**, *32*, 101904.

[6] D. Shah, F. S. Mjalli, Effect of water on the thermo-physical properties of Reline: An experimental and molecular simulation based approach*.* *Physical Chemistry Chemical Physics* **2014**, *16*, 23900-23907.

[7] Y. Zhou, J. Dong, P. Zhao, J. Zhang, M. Zheng, J. Feng, Imaging of Single Bacteria with Electrochemiluminescence Microscopy*.* *J. Am. Chem. Soc.* **2023**, *145*, 8947-8953.

[8] T. Yang, Y. Gao, Z. Liu, J. Xu, L. Lu, Y. Yu, Three-dimensional gold nanoparticles/prussian blue-poly(3,4-ethylenedioxythiophene) nanocomposite as novel redox matrix for label-free electrochemical immunoassay of carcinoembryonic antigen*.* *Sens. Actuator. B Chem.* **2017**, *239*, 76-84.

[9] S. Khumngern, N. Nontipichet, P. Thavarungkul, P. Kanatharana, A. Numnuam, Smartphone-enabled flow injection amperometric glucose monitoring based on a screen-printed carbon electrode modified with PEDOT@PB and a GOx@PPtNPs@MWCNTs nanocomposite*.* *Talanta* **2024**, *277*, 126336.

[10] J. B. Ayers, W. H. Waggoner, Synthesis and properties of two series of heavy metal hexacyanoferrates*.* *J. Inorg. Nucl. Chem.* **1971**, *33*, 721-733.

[11] L. Zhang, H. Peng, P. A. Kilmartin, C. Soeller, J. Travas-Sejdic, Poly(3,4-ethylenedioxythiophene) and Polyaniline Bilayer Nanostructures with High Conductivity and Electrocatalytic Activity*.* *Macromolecules* **2008**, *41*, 7671-7678.

[12] R. Barber, J. Davis, P. Papakonstantinou, Stable Chitosan and Prussian Blue-Coated Laser-Induced Graphene Skin Sensor for the Electrochemical Detection of Hydrogen Peroxide in Sweat*.* *ACS Appl. Nano Mater.* **2023**, *6*, 10290-10302.

[13] M. Cao, H. Wang, H. Tang, D. Zhao, Y. Li, Enzyme-Encapsulated Zeolitic Imidazolate Frameworks Formed Inside the Single Glass Nanopore: Catalytic Performance and Sensing Application*.* *Anal. Chem.* **2021**, *93*, 12257-12264.

[14] W. Yang, R. Xu, F. Mu, J. Chauvin, S. Cosnier, X.-J. Zhang, D. Shan, Vesicle-confined redox mediation via biomimetic ABTS-liposomes enables stable electron relay and dual-mode H2O2 detection*.* *Biosens. Bioelectron.* **2026**, *292*, 118075.

[15] U. Pinkernell, B. Nowack, H. Gallard, U. von Gunten, Methods for the photometric determination of reactive bromine and chlorine species with ABTS*.* *Water Res.* **2000**, *34*, 4343-4350.

[16] V. A. Zamolo, G. Valenti, E. Venturelli, O. Chaloin, M. Marcaccio, S. Boscolo, V. Castagnola, S. Sosa, F. Berti, G. Fontanive, M. Poli, A. Tubaro, A. Bianco, F. Paolucci, M. Prato, Highly Sensitive Electrochemiluminescent Nanobiosensor for the Detection of Palytoxin*.* *ACS Nano* **2012**, *6*, 7989-7997.

[17] Z. Tang, K. Wen, Y. Guo, P. Xie, K. Li, Y. Chen, J.-L. Liu, R. Yuan, K. Peng, An Efficient Luminol–H2O2 Electrochemiluminescence System with Porous Bimetallic Organic Gels as Signal Booster and Elaborate Heterosequence Aptamer as Recognition Component for Ultrasensitive Biosensing*.* *Anal. Chem.* **2025**, *97*, 10772-10781.
